# The characterization of a new set of EST-derived simple sequence repeat (SSR) markers as a resource for the genetic analysis of *Phaseolus vulgaris*

**DOI:** 10.1186/1471-2156-12-41

**Published:** 2011-05-09

**Authors:** Robertha AV Garcia, Priscila N Rangel, Claudio Brondani, Wellington S Martins, Leonardo C Melo, Monalisa S Carneiro, Tereza CO Borba, Rosana PV Brondani

**Affiliations:** 1 Universidade Federal de Goiás, Escola de Agronomia, CEP 74690-900, Goiânia, GO, Brazil; 2 Embrapa Arroz e Feijão, Rodovia GO-462, km 12 Zona Rural, CEP 75375-000, Santo Antônio de Goiás, GO, Brazil; 3 Universidade Federal de Goiás, Instituto de Informática, CEP 74690-815, Goiânia, GO, Brazil; 4 Universidade Federal de São Carlos, Centro de Ciências Agrárias, Via Anhanguera, km 174, CEP 13600-970, Araras, SP, Brazil

## Abstract

**Background:**

Over recent years, a growing effort has been made to develop microsatellite markers for the genomic analysis of the common bean (*Phaseolus vulgaris*) to broaden the knowledge of the molecular genetic basis of this species. The availability of large sets of expressed sequence tags (ESTs) in public databases has given rise to an expedient approach for the identification of SSRs (Simple Sequence Repeats), specifically EST-derived SSRs. In the present work, a battery of new microsatellite markers was obtained from a search of the *Phaseolus vulgaris *EST database. The diversity, degree of transferability and polymorphism of these markers were tested.

**Results:**

From 9,583 valid ESTs, 4,764 had microsatellite motifs, from which 377 were used to design primers, and 302 (80.11%) showed good amplification quality. To analyze transferability, a group of 167 SSRs were tested, and the results showed that they were 82% transferable across at least one species. The highest amplification rates were observed between the species from the *Phaseolus *(63.7%), *Vigna *(25.9%), *Glycine *(19.8%), *Medicago *(10.2%), *Dipterix *(6%) and *Arachis *(1.8%) genera. The average PIC (Polymorphism Information Content) varied from 0.53 for genomic SSRs to 0.47 for EST-SSRs, and the average number of alleles per locus was 4 and 3, respectively. Among the 315 newly tested SSRs in the BJ (BAT93 X Jalo EEP558) population, 24% (76) were polymorphic. The integration of these segregant loci into a framework map composed of 123 previously obtained SSR markers yielded a total of 199 segregant loci, of which 182 (91.5%) were mapped to 14 linkage groups, resulting in a map length of 1,157 cM.

**Conclusions:**

A total of 302 newly developed EST-SSR markers, showing good amplification quality, are available for the genetic analysis of *Phaseolus vulgaris*. These markers showed satisfactory rates of transferability, especially between species that have great economic and genomic values. Their diversity was comparable to genomic SSRs, and they were incorporated in the common bean reference genetic map, which constitutes an important contribution to and advance in *Phaseolus vulgaris *genomic research.

## Background

Since the beginning of civilization, the legumes, together with the cereals, have been considered essential for the development of modern agriculture, occupying the second rank in terms of planted area and production of grains. In the human diet, they are a valuable source of protein and are used as sources of edible oils and fodder plants for animals [1]. The Leguminosae family, among the superior plants, is considered the third largest family. It is composed of approximately 700 genera and 20,000 described species, of which approximately 60 are domesticated. The *Phaseolus *genus belongs to the subfamily Papilionoideae. It originates in the Americas and is comprised of more than 30 species, whereas only *Phaseolus vulgaris, Phaseolus lunatus, Phaseolus coccineus, Phaseolus acutifolius *and *Phaseolus polyanthus *are domesticated [2]. The *Phaseolus vulgaris *species (common bean) is cultivated and occupies 85% of the production area of all *Phaseolus *species in the world [3]. Among vegetables and grains, the common bean is the most important for direct human consumption on all continents [4].

The significant advances in the genomic resources of the last two decades, including reduction of costs and greater accessibility to molecular technologies, have allowed for improvements in the knowledge regarding the structure and organization of the genomes of different organisms [5]. Recently, important progress has been made on the genome sequencing of the legumes, including soybean (*Glycine max*), barrel medic (*Medicago truncatula*), and birdsfoot trefoil (*Lotus japonicus*), all of which are considered model organisms due to their biological functions, reduced genome sizes and economic importance [6]. Among these species, the soybean genome was the largest sequenced using a shotgun approach, and the assembled sequences were integrated in a genetic and physical map, allowing the investigation of the genome composition, duplication, organization and transcriptional factors [7]. As a result, there is a realistic possibility to apply the knowledge attained regarding the soybean genome to other economically important legume species, particularly due to the synteny and collinearity among these plants. Phylogenetic relationships within the legume family [8] are reflected in relatively high similarity, or synteny, at the genome level among the temperate climate legumes, including *Medicago *sp. and pea [9], or between the tropical climate legumes, such as the common bean and soybean [10].

Comparative genomic analyses are important strategies for attempting to understand the structural and functional aspects of genomes and have contributed to the elucidation of evolutionary and phylogenetic aspects between species [5]. Macro-synteny studies have enabled the investigation of gene order conservation through comparative mapping or *in silico *sequence homology analyses. The development of primers from expressed sequences that are available in public databases have made their use more accessible but are still restricted to those species for which the genome sequence information is available. Among the legumes, microsatellites have been developed for the lentil [11], common bean [12-15], *Vigna *[16], chickpea [17], *Medicago *[18] and soybean [19,20]. The transferability of these markers between species from the same genus, and even across genera in the same family, is possible in many cases. The conservation of microsatellite loci has been described for several plant species [21]. In legumes, studies have demonstrated that microsatellite markers were successfully amplified between species in the genus *Medicago *[22,18], and a high intra-specific transferability rate was observed in the genus *Vigna *[23]. In the genus *Phaseolus*, [24] a transferability rate of 50% in microsatellite markers was observed between the species *P. coccineus, P. polyanthus, P. acutifolius *and *P. lunatus*.

The common bean (*Phaseolus vulgaris *L.) is a predominantly self-pollinating diploid species with a genome size estimated at 637 Mbp [25] that is distributed among 22 chromosomes (n = 11). The first genetic map developed for the common bean was derived from the crossing of BAT93 × Jalo EEP558 (BJ), which was considered to be the core map for the common bean based on 194 RFLP markers and is called the Map Davis [26]. Later, using the same BJ mapping population, a second version of the map was developed that integrated RFLP and RAPD markers [27], followed by a more complete version that included 563 markers and covered 1,226 cM of genome length. In the present decade, with the availability of microsatellite markers for the common bean, an increasing set of these markers began to be integrated into the core map [13,28], resulting in a linkage map based exclusively on microsatellite markers for the BJ population [29]. More recently, a new, expanded version of the core linkage map, also using the BJ population, was released and included markers with putative gene functions [30].

The specific objectives of this work were the following: (1) to characterize the common bean EST databases for the presence and nature of microsatellites, (2) to develop a battery of new microsatellite markers for *Phaseolus vulgaris *from the *in silico *analysis, (3) to assess the polymorphism, diversity and degree of transferability across the Leguminosae family between a group of genomic-SSRs and EST-SSRs to establish a comparison among the results obtained and (4) to screen for polymorphic microsatellite markers in the BAT93 X Jalo EEP558 population for their integration into the core map.

## Results

### SSR amplification

A total of 9,583 valid ESTs corresponding to 3,281 contigs and 6,302 singletons were screened for the presence of useful SSRs. Based on the criteria adopted, 4,764 SSRs with lengths of at least six repeat units were identified, including 1,950 in contigs and 2,814 in singletons. The mononucleotide motifs were the most abundant (80%), followed by mono-compounds (15%), which contained more than two repeat motifs, dinucleotides (1.7%), trinucleotides (1.5%), di- (1.32%), tri-compounds (0.38%), hexanucleotides (0.06%) and tetranucleotides (0.04%). Among the dinucleotide sequences, the motif AG/TC had the highest frequency, representing 71% of the sampled sequences, followed by motifs AT/TA (20%), TG/AC (8%) and GC/CG (1%). A set of 377 EST-SSR markers were developed. Primer sequences and information on the SSRs derived from the EST GenBank are available in Additional file 1, Table S1.

The first evaluation was performed to check for primer amplification quality and performance under the same amplification conditions. The results demonstrated that out of the 377 EST-SSRs, 281 (74.5%) primers generated a scorable amplification product, while 24 (6.37%) generated non-specific products and 72 (19.1%) failed to amplify. In a second evaluation, after making adjustments to the PCR conditions, 30 primers were recovered, which increased the number of EST-SSRs that generated satisfactory amplification products to 302 (80.1%) (Additional file 1, Table S1). Of these, 149 (49.3%) showed single bands and 153 (50.6%) showed interpretable PCR products, although they contained some nonspecific bands. The remaining 33 (8.75%) primers failed to produce bands with a scorable pattern, even after adjustments to the PCR conditions, and were not used in additional analyses. Regarding the efficiency of amplification for each repeat motif, mononucleotides (34.5%) had the highest rate of PCR amplification, followed by di- (31.3%), tri- (22.3%), hexa- (0.53%) and tetranucleotides (0.26%). Among the 302 useful EST-SSRs markers, 232 (76.8%) generated amplification products with the expected size, while 48 (15.9%) and 22 (7.28%) markers generated products that were longer and smaller than the expected size, respectively.

From the 44 SSRs previously developed from genomic libraries and based exclusively on dinucleotide repeats, 13 (29.5%) were successfully amplified with a clear and specific band pattern, five (11.4%) with interpretable PCR products but containing some stutter bands; the remaining 26 (59.1%) failed to amplify and were not used in further analyses. Thus, a total of 315 markers (302 EST-derived and 13 genomic SSRs) that showed a scorable pattern of amplification products were screened for polymorphism in the BJ population.

### Transferability across the Leguminosae and within the genus *Phaseolus*

To assess the transferability of the SSR loci across Legume species, the cross-amplification of 167 primers (102 genomic SSRs, five previously developed EST-derived markers, and 60 newly developed EST-SSR markers that showed robust amplification products) were tested against 20 genotypes, representing 11 species of the Leguminosae family. These species included the following: *Phaseolus vulgaris, Medicago sativa, Phaseolus lunatus, Phaseolus coccineus, Phaseolus acutifolius, Vigna mungo, Vigna angularis, Vigna unguiculata, Glycine max, Arachis hypogaea *and *Dipteryx alata*. The resulting amplification profiles for each locus were assigned individually for each species and are shown as Additional file 2 (Table S2). From the 65 EST-SSRs, 61 (93.8%) amplified across at least one species, and only four (6.15%) were species-specific. For the 102 markers that were derived from genomic libraries, 76 (74.5%) amplified across at least one species and 26 (25.5%) failed to produce any cross-amplification product (Additional file 3, Figure S1). The number of transferable markers across all species ranged from 119 (71.3%) to three (1.79%) for *Phaseolus acutifolius *and *Arachis hypogaea*, respectively, with an average cross-amplification ratio of 32%.

As expected, the highest ratio of interspecies cross-amplification was observed for the species within the genus *Phaseolus *(63.7%), followed by *Vigna *(25.9%), *Glycine *(19.8%), *Medicago *(10.2%) and *Dipterix *(6%), while the genus *Arachis *had a transferability ratio of only 1.8%. Among the entire set of markers, 69 (41.3%) were transferable across the three species within the genus *Phaseolus*, from which 45 (69.2%) were EST-derived markers and 24 (23.5%) were genomic markers. From the whole set of transferable SSRs, the best pattern of amplification was observed for those that were transferred to the genus *Phaseolus acutifolius *(119 markers), of which 60 (50.4%) were EST-SSRs and 59 (49.6%) were genomic SSRs.

The molecular sizes of the alleles produced by each SSR marker are shown in Additional file 2, Table S2. Among the 137 transferable primer pairs, 108 produced amplification products within the expected size range, 16 produced products that were considered longer than expected and 13 produced products that were smaller than expected. Markers PVEST112 and PVEST6 produced alleles that were 182 bp longer and 61 bp smaller, respectively, than the expected size ranges.

### Genetic diversity of EST and genomic SSRs

The genetic diversity of SSRs was assessed based on the 16 *Phaseolus vulgaris *genotypes, which represent six commercial grain classes. Among the entire set of 167 SSRs tested, 121 (72.5%) detected allelic variation between the genotyped samples, and the remaining 46 (27.5%) were monomorphic. Hence, the estimates described below were obtained only for the polymorphic markers, from which 53 were EST-SSRs and 68 were genomic SSR markers. The average PIC value for the complete set of markers was 0.50, with estimates ranging from 0.12 (PVEST203) to 0.89 (BM187). With regard to the 68 genomic SSRs, the number of alleles ranged from a minimum of two to a maximum of 11 alleles, with an average of four alleles per locus. The PIC values ranged from 0.22 to 0.89, with an average of 0.53. Among the EST-SSRs, the minimum and maximum numbers of alleles were two and 11, respectively, and the average number of alleles per locus was three. The mean PIC value was estimated to be 0.47, ranging from 0.12 to 0.85. The detailed results are presented in Tables 1 and 2.

**Table 1:** Genetic characterization of the 102 genomic SSRs in common beans

Locus	***p***^**(1)**^	*N*a	*Ho*	PIC	**Transf. species**^**(2)**^
AG1	0.563	5	0.563	0.551	2,3,4,5,6, 7
AJ416389	0.714	3	0.000	0.406	-
AJ416391	0.667	2	0.000	0.346	-
AJ416395	-	-	-	-	-
BM003	-	-	-	-	2, 4
BM006	-	-	-	-	3, 4
BM016	-	-	-	-	1,2,3, 4
BM020	-	-	-	-	-
BM068	0.500	3	0.000	0.456	2, 4
BM098	0.688	2	0.000	0.337	1,2,3,4,5,6,7,8,9, 10
BM114	0.538	4	0.000	0.576	-
BM137	0.200	9	0.000	0.857	3
BM138	0.571	2	0.000	0.370	-
BM140	0.875	3	0.000	0.215	1,2, 4
BM142	-	-	-	-	2,3,4, 7
BM143	0.200	10	0.000	0.868	2, 4
BM146	-	-	-	-	2,3,4,5, 7
BM148	-	-	-	-	2,3,4, 7
BM149	0.813	2	0.000	0.258	2,4,5, 6
BM151	0.600	3	0.000	0.499	2,3, 4
BM153	-	-	-	-	-
BM154	0.250	9	0.000	0.835	2, 4
BM155	0.667	3	0.000	0.438	4
BM157	0.733	2	0.000	0.315	1,2,3,7, 8
BM158	0.417	6	0.000	0.719	3
BM159	0.625	3	0.000	0.468	4
BM160	0.625	6	0.000	0.553	2, 4
BM161	-	-	-	-	2,3,5,6, 7
BM164	0.688	3	0.000	0.427	2,3,4,7, 10
BM165	0.667	2	0.000	0.346	-
BM166	-	-	-	-	2,4, 5
BM167	0.500	3	0.000	0.511	3,4, 7
BM175	0.667	4	0.000	0.462	4, 5
BM181	0.500	2	0.000	0.375	2, 4
BM183	0.438	5	0.000	0.657	2,3,4
BM184	0.571	3	0.000	0.501	-
BM185	0.313	4	0.000	0.675	1,2,3,4,5,7, 8
BM187	0.125	11	0.000	0.889	1,2,3, 4
BM189	0.500	4	0.000	0.570	2, 4
BM197	0.625	3	0.000	0.468	2,3,4,5,6,7, 8
BM200	0.286	7	0.000	0.768	2, 4
BM201	0.375	4	0.000	0.658	2, 4
BM202	0.714	2	0.000	0.325	2, 3, 4
BM205	0.375	5	0.000	0.711	-
BM210	0.385	4	0.000	0.613	2, 4
BM211	0.286	7	0.000	0.792	2, 4
BM212	0.444	4	0.000	0.607	-
GATs11B	0.625	2	0.000	0.359	4
GATs54	0.571	2	0.000	0.370	2
PV005	0.375	6	0.000	0.712	-
PV011	0.375	4	0.000	0.636	2, 4
PV012	0.688	2	0.000	0.337	2, 4
PV013	0.462	7	0.000	0.709	-
PV025	0.200	7	0.000	0.825	2, 4
PV035	0.600	5	0.000	0.562	2, 4
PV038	0.500	2	0.000	0.375	-
PV051	-	-	-	-	2,3,4,5, 7
PV053	0.733	3	0.000	0.388	2,3, 4
PV055	0.367	5	0.467	0.699	2, 4
PV060	0.364	6	0.000	0.726	2,3, 4
PV067	0.400	4	0.000	0.624	-
PV077	0.800	3	0.000	0.309	2,3, 4
PV080	-	-	-	-	1,2,3, 4
PV087	0.625	5	0.000	0.539	2, 4
PV096	-	-	-	-	3
PV097	0.333	5	0.000	0.738	2,3,5,6, 7
PV101	-	-	-	-	8
PV102	0.462	3	0.000	0.566	-
PV105	-	-	-	-	-
PV112	0.625	3	0.000	0.428	2,3, 4
PV113	0.533	3	0.000	0.456	2, 4
PV118	0.688	2	0.000	0.337	2, 4
PV131	-	-	-	-	4
PV140	-	-	-	-	2,3,4,5,7,8, 10
PV148	-	-	-	-	3
PV162	-	-	-	-	2
PV163	0.333	7	0.000	0.775	2 e 4
PV168	0.667	2	0.000	0.346	2, 4
PV169	0.455	4	0.000	0.623	-
PV174	-	-	-	-	2 e 4
PV180	-	-	-	-	2, 4
PV193	0.500	3	0.000	0.544	
PV194	-	-	-	-	1,2, 3
PV198	0.583	2	0.000	0.368	1,2, 3
PV200	-	-	-	-	-
PV202	-	-	-	-	2,3,4, 8
PV204	-	-	-	-	3
PV207	-	-	-	-	3
PV215	-	-	-	-	-
PV221	-	-	-	-	-
PV231	-	-	-	-	2, 4
PV237	0.844	2	0.313	0.229	3
PV243	-	-	-	-	2,3,4,5, 7
PV251	-	-	-	-	1,2,3,4, 7
PV254	-	-	-	-	-
PV258	0.813	2	0.375	0.258	4
PV259	0.500	2	0.000	0.375	-
PV265	0.813	2	0.000	0.258	-
PV268	-	-	-	-	-
PV270	0.636	2	0.000	0.356	2, 4
PV272	0.250	8	0.000	0.825	3
PV243	0.250	7	0.000	0.812	-

Detailed information of the allele frequency (*p*) average over locus, the number of alleles per locus (*N*a), the observed heterozygosity (*Ho*), the polymorphism information content (PIC) and the species for which the SSRs were transferable.

^(1)^Average allele frequency

^(2) ^Transferable species: 1- *Medicago sativa*; 2- *Phaseolus lunatus; *3- *P. coccineus; *4- *P. acutifolius*; 5- *Vigna mungo*; 6- *V. angularis*; 7-*V. unguiculata*; 8- *Glicine max*; 9- *Arachis hypogaea*; 10- *Dipteryx alata*.

**Table 2:** Genetic characterization of 65 EST-SSRs in common beans

Locus	***p***^***(1)***^	*Na*	*Ho*	PIC	**Transf. species**^**(2)**^
BMc78	0.594	5	0.063	0.551	2, 4
BMd64-1	-	-	-	-	1,2,3, 4
PVEST001	0.429	3	0.000	0.567	
PVEST006	0.313	8	0.000	0.780	2,3,4, 7
PVEST007	0.625	4	0.000	0.524	-
PVEST008	0.375	7	0.063	0.715	3,4,5,6,7,8, 9
PVEST010	0.750	2	0.000	0.305	2,3,4,5,7, 8
PVEST017	0.267	6	0.067	0.779	1,2,3,4,5,7, 8
PVEST023	0.600	2	0.000	0.365	2,4, 8
PVEST026	0.433	3	0.067	0.572	2,3,4,5, 7
PVEST029	0.733	3	0.000	0.370	2, 4
PVEST030	0.313	5	0.000	0.717	2,3,4,5,7,8,9, 10
PVEST034	0.625	3	0.000	0.428	2,3,4,6, 7
PVEST042	0.333	6	0.067	0.727	-
PVEST049	0.600	3	0.000	0.440	2,4, 5
PVEST055	0.688	4	0.000	0.458	2,3,4,5,6, 7
PVEST061	0.625	2	0.000	0.359	2,3,4, 5
PVEST071	0.625	4	0.000	0.483	2,3,4, 7
PVEST072	0.267	8	0.000	0.827	2,4, 8
PVEST073	0.625	2	0.000	0.359	2,4,5,7,8, 10
PVEST086	0.688	2	0.000	0.337	2,3,4,5,6,7, 8
PVEST098	0.406	4	0.063	0.602	2,4,5, 7
PVEST099	0.813	2	0.000	0.258	2,3,4,5,6,7 e 8
PVEST101	0.688	3	0.000	0.398	2,3,4,6, 7
PVEST106	-	-	-	-	1,2,3, 4
PVEST107	-	-	-	-	1,2,3,4,5,6,7, 8
PVEST112	-	-	-	-	2,3,4, 7
PVEST114	0.625	2	0.000	0.359	2,4,5
PVEST120	-	-	-	-	2,3, 4
PVEST121	0.786	2	0.000	0.280	2,3 4,5, 8
PVEST127	-	-	-	-	1,2,3,4,5,6,7,8, 10
PVEST137	0.219	11	0.188	0.851	2,3,4,5,6,7, 8
PVEST138	-	-	-	-	2,3,4,5,7, 8
PVEST144	-	-	-	-	2, 4
PVEST147	0.433	3	0.067	0.572	2,3, 4
PVEST161	-	-	-	-	2,3,4, 8
PVEST164	0.500	3	0.000	0.511	2,3,4,5,6, 7
PVEST166	0.438	4	0.000	0.612	2,3,4,7, 8
PVEST168	-	-	-	-	2,3,4,5,6, 7
PVEST186	0.600	3	0.000	0.485	2,4, 8
PVEST195	-	-	-	-	1,2,3,4,5, 7
PVEST196	0.688	3	0.000	0.398	2,3,4, 5
PVEST197	-	-	-	-	1,2,3,4,7,8, 10
PVEST203	0.929	2	0.000	0.124	-
PVEST217	0.500	3	0.000	0.544	2,3,4,5,7,8, 10
PVEST221	0.625	2	0.000	0.359	3,4, 5
PVEST232	0.688	2	0.000	0.337	2,3,4,6, 7
PVEST233	0.625	3	0.000	0.482	2,4, 5
PVEST234	0.625	2	0.000	0.359	2,3,4,5,7, 8
PVEST249	0.625	2	0.000	0.359	-
PVEST251	0.667	2	0.000	0.346	2,3,4,8,9, 10
PVEST258	0.286	7	0.000	0.805	2,3, 4
PVEST259	0.625	3	0.000	0.428	2,3,4, 5
PVEST260	0.438	3	0.000	0.575	2,3,4,5,6,7, 8
PVEST271	0.625	2	0.000	0.359	2,4,6, 7
PVEST272	0.533	3	0.000	0.509	2,3,4,5,7, 8
PVEST293	0.563	2	0.000	0.371	2,3,4
PVEST304	0.467	3	0.000	0.556	2, 4
PVEST320	0.625	2	0.000	0.359	2,3,4,5,6,7, 8
PVEST336	0.563	2	0.000	0.371	2,3,4,5,6,7, 8
PVEST359	0.688	2	0.000	0.337	2,4
PVEST368	0.688	2	0.000	0.337	2,3,4,5,6, 7
X04001	0.643	3	0.000	0.464	1,2,3,4,5, 7
X13329	0.625	3	0.000	0.468	3,4,5, 7
X60000	0.750	3	0.000	0.354	2, 4

Detailed information of the allele frequency (*p*) average over locus, the number of alleles per locus (*N*a), the observed heterozygosity (*Ho*), the polymorphism information content (PIC) and the species for which the SSRs were transferable.

^(1)^Average allele frequency

^(2) ^Transferable species: 1- *Medicago sativa*; 2- *Phaseolus lunatus; *3- *P. coccineus; *4- *P. acutifolius*; 5- *Vigna mungo*; 6- *V. angularis*; 7-*V. unguiculata*; 8- *Glicine max*; 9- *Arachis hypogaea*; 10- *Dipteryx alata*.

### Linkage analysis

From the 315 new SSRs screened for polymorphism, 76 (24.1%) segregated in the BJ population, of which 72 were EST-SSRs and four were genomic SSRs. Only a set of two (2.63%) SSR markers could be easily interpretable in agarose gels (PVEST1 and PVEST279), while the remaining 74 (97.4%) were genotyped in polyacrylamide gels (Additional file 4, Figure S2). As indicated by the χ^2 ^test, 54 (71.1%) of the 76 SSRs segregated in accordance with the expected Mendelian ratios, while 22 (28.9%) showed significant segregation distortion at p < 0.05. Using the FDR method to correct for multiple comparisons by controlling the chance of a false positive, only 12 (15.9%) markers showed significant segregation distortion. Among these, four were skewed towards the Andean parent, Jalo EEP558, and eight were skewed towards the Mesoamerican parent, BAT93. All loci with segregation distortions were EST-SSRs and were maintained in the linkage analysis because no order alteration was observed when they were removed from the analysis. The 12 distorted markers were mapped across four linkage groups (chromosomes 1, 2, 3 and 7), and two clusters of four and three markers were observed on chromosome 7B. All of the distorted markers that were mapped to chromosome 7 showed distortions towards BAT93, and three out four markers that were skewed towards EEP558 were mapped to chromosome 1. Despite the distortions, all mapped loci were placed with a minimum LOD score of 3.0.

**Figure 1:**
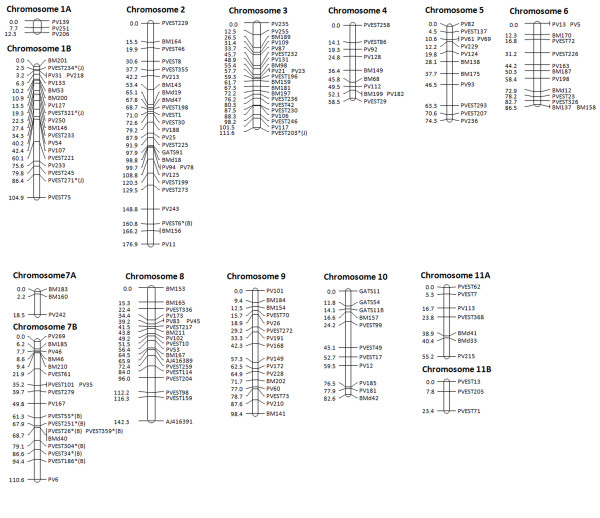
**Common bean linkage map based on 182 SSR markers**. The mapped markers include genomic and EST-derived SSR loci segregating in the BAT93 × Jalo EEP558 population. Distances are indicated in cM Kosambi. Microsatellites were mapped with a minimum LOD of 3.0. Markers that showed segregation distortions are starred (*; p < 0.05) and labeled with (B) or (J), indicating the predominance of alleles from BAT93 or Jalo EEP558, respectively.

The integration of the SSRs into the BJ linkage map resulted in a dataset of 199 polymorphic markers, from which 117 were genomic SSRs and 82 were EST-SSRs. A total of 182 (91.5%) markers could be mapped at an LOD score ≥3.0 (θ = 0.30) and were distributed into 14 linkage groups. These groups represent the 11 chromosomes of the common bean and the three extra small chromosome segments (Figure 1). The extra chromosomes (1A, 7A and 11B) had three markers each and are unlinked segments mapped to the tip of chromosomes 1 and 7 and the end of chromosome 11. Chromosome assignments were performed according to [31]. Marker distribution along the chromosomes ranged from 3 (chromosomes 1A, 7A and 11B) to 26 (chromosome 2), and chromosome sizes varied from 12.33 cM (chromosome 1A) to 176.72 cM (chromosome 2) with an average size of 88.82 ± 44.07 cM. The total map distance was estimated at 1,156.2 cM with an average marker distance of 6.65 ± 1.64 cM, and the maximum distance between two markers was 26.0 cM (PVEST159 and AJ416391 on the end of chromosome 3). The distribution of the EST-SSRs appeared to be relatively random and dispersed throughout the *Phaseolus *genome. Among the 72 polymorphic EST-SSRs, 65 were assigned to 12 chromosomes with the number of markers ranging from 3 (chromosomes 11A, 11B, 5, 3, 8 and 9) to 11 (chromosome 2).

The comparative analysis was performed using the current map and a previous map, which was also based solely on SSR markers and on the same BJ population developed by [29] and considering the chromosome assignments as defined by Pedrosa-Harand et al. 2008 [31]. From a set of 123 SSR markers common to both segregation analyses, 105 were mapped to the current linkage analysis, while 106 were mapped to the previous map. The marker BM152, which was mapped to chromosome 9 in the previous study, could not be mapped in our study. Based on a visual comparison, it was observed that all SSRs common to both maps maintained their positions in the same linkage group. However, order inversions were observed in almost all chromosomes, except for chromosome 11. All inversions observed were due to single marker rearrangements, which accounted for 19 order inversions and resulted in a marker order conservation of 81%.

To explore the potential of the EST-SSRs for use in the structural genome exploitation of *Phaseolus*, a search was performed in GenBank, using BLASTX, against the sequences of the polymorphic markers. The BLAST results showed that 44 (57.9%) of the 76 polymorphic EST-SSRs had similarity to GenBank sequences, while the other 32 (42.1%) did not show any significant matches (Table 3). Out of the 44 EST-SSRs, 13 (29.5%) showed homology with genes of known putative functions in the Legume family and 31 (70.5%) with different organisms, predominantly with *Arabidopsis thaliana*.

**Table 3:** Putative functions of polymorphic EST-SSR markers, as determined by BLASTX

Locus	Chrom	**Sequence ID**^**1**^	Top E-value	Species	Putative gene function
PVEST008	-	ALV_007_B10_G1	7E-40	A. thaliana	Lsd One Like 1
PVEST026	7B	ALV_012C_E03_B1	1E-35	V. vinifera	Hypothetical protein
PVEST029	4	ALV_013C_A09_B1	7E-23	P. vulgaris	Ribulose bisphosphate carboxylase
PVEST042	3	ALV_014C_G08_B1	4E-67	G. max	Plastid 3-keto-acyl-ACP synthase II-B
PVEST049	10	ALV_015A_G09_B1	1E-104	M. truncatula	Cytochrome P450 monooxygenase
PVEST061	7B	ALV_017A_E11_B1	1E-51	P. sativum	DNA-binding protein DF1
PVEST071	11B	Contig1282	7E-35	PVPR3	PVPR3
PVEST072	6	Contig1299	1E-52	M. truncatula	Ferric reductase-like transmembrane component
PVEST073	9	Contig1338	1E-105	G. hirsutum	Small GTPase
PVEST086	4	Contig1621	4E-18	V. vinifera	Hypothetical protein
PVEST099	10	Contig1916	2E-39	M. truncatula	Protein of unknown function DUF581
PVEST101	7B	Contig1984	8E-13	P. sativum	Drm4
PVEST114	8	Contig2442	1E-92	A. thaliana	NLI interacting factor (NIF)
PVEST134	-	Contig2970	1E-137	P. montana	Isopentenyl pyrophosphate isomerase
PVEST147	-	Contig03274	2E-83	A. thaliana	Constitutive Disease Resistance 1
PVEST155	-	Contig0520	4E-61	A. thaliana	Sterol-C-methyltransferase
PVEST158	-	Contig0551	9E-20	A. thaliana	Unknown protein
PVEST166	-	Contig0802	1E-32	A. thaliana	kinesin-like protein
PVEST176	-	LVS_010_H04_b2	6E-46	A. thaliana	Postsynaptic protein-related
PVEST186	7B	LVS_040_F02_b1	2E-90	V. vinifera	Hypothetical protein
PVEST188	-	LVS_042_C11_b1	3E-19	A. corniculatum	Aquaporin 1
PVEST198	2	NOD_217_A08_053	3E-36	A. thaliana	Leucine-rich repeat family protein
PVEST204	8	NOD_223_E06_040	1E-68	A. thaliana	Hypothetical protein
PVEST205	11B	NOD_225_C03_019	3E-58	A. thaliana	Unnamed protein product
PVEST207	5	NOD_230_E11_084	2E-24	A. thaliana	ATOFP14/OFP14 - ovate family protein 14
PVEST227	-	POD_036_B08_061	4E-35	M. truncatula	Hypothetical protein MtrDRAFT_AC160516g6v2
PVEST229	2	POD_039_G09_068	3E-61	A. thaliana	Putative ABC transporter
PVEST230	3	POD_040_B03_025	5E-23	A. thaliana	Putative ATPase (ISW2)
PVEST232	3	PVEPSE2003D01	5E-25	A. thaliana	Unknown protein
PVEST245	1B	PVEPSE2022F10	1E-62	V. unguiculata	Patatin-like protein
PVEST249	-	PVEPSE2030D05	1E-13	M. truncatula	Alpha-6-galactosyltransferase
PVEST251	7B	PVEPSE2032A08	1E-24	A. thaliana	Zinc Finger Protein 8
PVEST253	-	PVEPSE3007B09	1E-17	P. vulgaris	Cytochrome f
PVEST260	-	PVEPSE3030P01	3E-71	P. vulgaris	DnaJ-like protein
PVEST289	-	Contig0145	7E-45	R. palustris	Expansin 11 precursor
PVEST292	-	Contig1625	1E-104	M. truncatula	CBS
PVEST293	5	Contig1723	1E-99	R. communis	NADP-dependent malic protein
PVEST304	7B	Contig2620	3E-78	A. thaliana	Unknown protein
PVEST308	-	Contig3013	1E-121	V. radiata	ARG10
PVEST321	1B	Contig0673	2E-19	V. vinifera	Hypothetical protein
PVEST323	-	Contig0073	5E-41	V. vinifera	Hypothetical protein
PVEST326	6	Contig0863	5E-79	L. esculentum	Type 5 protein serine/threonine phosphatase 62 kDa isoform
PVEST336	8	ALV_011A_G01_B1	4E-53	A. thaliana	ATNSI (Nuclear Shuttle Interacting)
PVEST355	2	NOD_243_H08_064	6E-65	M. truncatula	Protein of unknown function DUF6, transmembrane

List of SSR markers indicating the position on the *Phaseolus vulgaris *linkage map, the sequence identity from which the SSRs were derived and the BLASTX results.

^1^For additional details, see the references [30] and [50].

## Discussion

In the present work, the distribution of repetitive sequences, or microsatellites, formed by either one or more base pairs of longer than six units, has been studied to develop a broad set of useful SSR markers for the common bean derived from a public EST database. The goal was to attribute a genetic value to the EST-SSRs to increase their potential use. In addition, the relatively low cost to obtain these markers, when compared to the development of genomic libraries, is an attractive choice. This choice is evident mainly in species with a narrow genetic base for which a high number of SSRs is necessary to detect polymorphism. Currently, there are about 36,626 sequences in the *Phaseolus *EST data set, which accounts for an increase of 31% in the last six months and represents a new potential source of SSR markers for the common bean. Similar approaches for SSR mining in EST databases have been applied for the common bean [13,15] as were used for the other related species of the Legume family [6,32]. From our data mining, the criteria established for SSR identification was unusual and less stringent, leading to the identification of mononucleotide and compound repeats. As a consequence, mononucleotides were the most frequent, followed by di- and trinucleotides. This result is in contrast with the previous observations for several species [33], but it is consistent with the observations [34] for dicotyledons. The composite motifs accounted for 30% of the SSRs identified, considering dinucleotide repeats or higher motifs, which was similar to the findings reported by [15] that identified 34% of such motifs. A reduced set of available markers would result from screening the non-redundant *Phaseolus *ESTs for SSRs, using the parameters of dinucleotide or more repeats, limiting the length of SSRs and keeping only the perfect repeats. Consequently, only 3.27% of the sequences containing SSRs would be adequate for primer design, which translates to 156 EST-SSRs. Indeed, in *Arabidopsis *and pepper, very close estimates were obtained when the criteria of stringency was increased, resulting in 3% and 3.6% of ESTs containing SSRs, respectively [33,35].

Although most markers were based on mononucleotide repeats, they showed satisfactory levels of PCR amplification that were even higher than those derived from the genomic sequences of common beans. The search for these repeats increased the number of SSR markers available for genetic analysis with a broad spectrum of applications. The rates of SSR amplification normally show a wide range in plants, such as those reported for barley and tomato with 64% and 83%, respectively [36,37]. In the present work, 80% of the EST-SSRs were amplified. Similar levels of EST-SSR amplification have been reported for the common bean by [12] and [13]. These reports also found successful amplification in 81% of the SSRs derived from the public database GenBank. A slightly higher value of 87% was reported by [15] using EST-SSRs derived from a private database. These values are comparable to those reported for SSRs derived from genomic libraries, which are described by [29] and [38], who found amplification rates of 86% and 81%, respectively. The high rate of success in the amplification of EST-SSRs in the common bean may be the result of several factors, such as the quality of the sequences from which the primers were derived, the adequate criteria used for primer design and the use of the same species for the design and amplification of the primer set. Although EST-derived SSR markers are generally less polymorphic than genomic SSRs, the value of EST-SSRs when compared to genomic SSRs is enhanced by several factors. These factors include their level of transferability, their potential to attribute function to genes affecting traits of interest and the readiness in the identification of SSRs by *in silico *data mining with reduced time, labor and cost.

Not surprisingly, the results presented in this work indicate that the EST-SSRs showed higher rates of transferability across the Legume species than the genomic SSRs. A total of 93.8% of the EST-SSRs were transferable for at least one species as compared with 74.5% of the genomic SSRs. The higher transferability rate of EST-SSRs can be explained by their correspondence to the transcribed component of a gene unit, which confers a high potential for inter-specific transferability [39]. The recent increase in the availability of the EST-SSRs derived from public databases can be expected to provide an additional source of transferable markers among less related species. Transferability of EST-SSRs has been reported for several species [37,40]. Transferability across the Legume family was lower than within *Phaseolus *genus, in which 64% of the markers produced amplification products in all tested species. These findings are in accordance with the report by [32], which described that a high degree of genome conservation has been identified between the model legumes *Medicago truncatula *and *Lotus japonicus*. However, genome conservation tends to be reduced as we move to the Phaseoloid clades, such as soybean (*G. max*), common bean (*P. vulgaris*) and *Vigna *(including cowpea and Asian *Vigna*). Generally, successful cross-amplification between the genera appears to be lower than within the genera. In a study with soybean, [41] found an amplification rate that ranged from 3% to 13% among the Legume genera, whereas for species within the genus, the level of transferability was up to 65%. As for the *Medicago *genus, a rate of 81% of the SSR markers tested was found to be transferable among *M. sativa *and *M. truncatula *[22]. Within the *Vigna *genus, [23] reported levels of SSR transferability that reached 90% among *V. umbellate *and *V. angularis*, whereas for the species *V. mungo, V. radiata *and *V. aconitifolia*, the amplification decreased to rates of 67% and 73%. In our study, the transferability of SSR markers between *P. vulgaris *and *G. max *was 10%, whereas most of the transferred markers (70%) were EST-SSRs. Because these two species are considered the most important cultivated legume in the world with a wide volume of available genetic linkage map information, the transferability of EST-SSRs may prove very useful for studies involving comparative genomics because of ability for information exchange.

Although the conserved nature of EST-SSRs facilitated transferability, these markers are considered less polymorphic than other sources of SSRs. In the current study, the level of polymorphism of the EST-SSRs (0.47) was slightly lower than that of SSRs derived from genomic libraries (0.53), with a mean number of three and four alleles, respectively. Similar results were found by [15] who obtained a PIC value of 0.44 and an average number of 2.7 alleles for EST-SSRs and a PIC value of 0.45 and an average number of 2.4 alleles per locus for genomic markers. High levels of polymorphism for genomic and EST-SSRs have been reported, where these markers were associated with an average number of 6 and 9.2 alleles per primer pair, respectively [28]. In the present study, 34 of the 167 loci were previously characterized using a more diverse set of sampled individuals, including accessions from Andean and Mesoamerican gene pools [14,28], to determine genetic diversity. The average PIC values, which reflect the allelic diversity and frequencies among the sampled individuals, observed for these markers were higher (0.70) than those found in the present work (0.50); these data provides strong evidence that the number and genetic relationship of the individuals used to access SSR genetic information could influence the estimates obtained. The correlation between PIC values and the index of transferability of the SSR markers was not observed. The loci that showed a greater rate of transferability were identified as monomorphic or had low PIC values. These findings are consistent with the theory that conserved genomic sequences are less variable.

Despite the reduced level of polymorphism rates found for the EST-SSRs, these markers were very useful for the genetic mapping of the BJ population, helping to increase the map coverage in the *Phaseolus *genome. Across the entire set of EST-SSRs tested for polymorphism in BAT93 and JALO EEP558, 24% segregated and were integrated into the BJ framework. Previous data indicated that genomic SSRs were almost two-fold more polymorphic than the EST-SSRs [29]. In a similar study, also using the BJ population, the polymorphism level of the EST-SSRs was significantly higher (51%) than the EST-SSR markers developed in our study [13]. The level of polymorphism of the EST-SSR markers could be attributed to the length and the number of repeat units and the SSR position inside the transcribed sequence. There is currently a great volume of genetic information derived from genome sequencing, high coverage genetic maps, BAC libraries and physical maps [42]. This increase in genetic information has allowed for a better understanding and has provided new insights into the elucidation of mechanisms involved in the expression of target traits and the transfer of knowledge from one species to another. Comparative genomics to assess synteny can facilitate the reciprocal use of genomics resources between different legume species, making the research cost-effective, efficient and useful for crop breeding [43].

Regarding the segregation distortion, 12 markers (15.9%) out of the 72 polymorphic EST-SSRs showed a significant deviation from the expected Mendelian segregation as shown by the FDR analysis (p < 0.05). The nature of the EST-SSRs could explain the high level of segregation distortion found in our study. A more extreme deviation in coding regions is expected because they are more susceptible to evolutionary pressures than non-coding regions. These markers were preferentially mapped on chromosomes 1 and 7 with a clear distinction between the markers that showed segregation distortions towards BAT93 or Jalo EEP558. The markers that were skewed towards the parent BAT93 were mapped on chromosome 7, whereas the markers that were skewed towards Jalo EEP558 were mapped on chromosome 1. The same pattern was observed in two previous studies using the same BJ mapping population [29,44]. The genes related to the domestication syndrome were mapped on chromosome 2 in two different studies [45,46]. This result establishes the hypothesis that the segregation distortion may have a biological basis on these chromosomes, and the parents, BAT93 and Jalo EEP558, may have suffered preferential selection on the specific regions of chromosomes 1 and 7 that resulted in the observed segregation distortion [47].

In our study, all markers were placed into 14 linkage groups, which exceeded the number of common bean chromosomes by three (n = 11). An increase in the number of markers and population size, which is currently formed by 76 individuals, would allow these three small linkage groups to integrate into their respective chromosomes. The total SSR map length (1,156.2 cM) was consistent with previously developed maps. Freyre et al. 1998 [44] reported a total map distance of 1,226 cM using the same BJ mapping population, and [48] reported a map size of 1,401 cM using a mapping population derived from the parents "Carioca" and "Guanajuato 31". However, an increase of almost 50% was observed in the SSR map length when compared to the SSR based map developed by [29] (606.8 cM). Because the sample size and mapping generation were the same, this variation in the estimation of the genome size can obviously be attributed to the higher marker density of the current map. In addition, [33] suggests that the EST-SSR-based linkage maps are expected to be larger than those based solely on anonymous SSRs because the recombination event may be more frequent in gene-rich regions than in non-coding regions. The genomic distribution of EST-SSR markers in this study was random and quite similar to the genomic SSR markers, providing a good coverage of all linkage groups.

A total of 207 SSR markers have been mapped on the BJ population to date, including 15 mapped by [12], 22 by [13], 94 by [29] and a new set of 76 SSRs in the present study, of which 74 were EST-derived markers. An additional effort was made in the last two years by Embrapa Rice and Beans to increase and make available the number of RILs derived form BJ population, which presently has 75 individuals [26], to achieve higher precision in the clustering and order analysis. In addition, the number of mapped markers tends to rapidly increase due to the continuously growing number of EST sequences that are becoming available for the common bean on public databases; this increased availability will contribute to the development of more EST-SSR-based markers and to the construction of more informative linkage maps. EST-SSR markers have the potential to greatly increase the degree of information provided by linkage maps because they can be readily associated with genes of known or putative functions, allowing for the direct association of markers in the map with quantitative traits of interest. Our results show that more than half (57.9%) of the EST-SSRs sequences were associated with sequences of putative genes in the GenBank, demonstrating the potential of these markers to be used for the genomic exploitation of the common bean. In addition, we demonstrated that EST-SSRs can be easily transferable among the Legume genus with levels of genetic information content comparable to those of genomic SSRs, which will contribute to expand their use in genetic analyses of *Phaseolus vulgaris*.

## Conclusions

A set of 377 EST-SSR markers were developed, from which 302 that showed good amplification quality are available for genetic analysis of *Phaseolus vulgaris*. These markers showed high rates of transferability, making them suitable for use in other economically important legume species. In addition, their genetic diversity was comparable with genomic SSRs, showing that they can be used in the genetic characterization of the common bean and related species. Finally, the newly developed EST-SSRs were incorporated into the BJ reference map, bringing a great contribution to the genetic dissection of *Phaseolus vulgaris*.

## Methods

### Data mining for EST sequences containing SSRs

The EST sequences were obtained from the "*Phaseolus vulgaris *EST Project site" (http://www.ccg.unam.mx/phaseolusest/), where a combined set of ESTs [49,50] was assembled [51] and made available. A standard PC (Intel P4 2 GHz processor, 512 KB cache, 512 MB RAM memory, 40 GB IDE hard disk) was used to process the data. The sequences were processed according to the methodology described in [52]. This method involved a search for SSRs using the TROLL [53] module with the Staden Package [54], followed by the use of a program that works with Primer3 [55] to design primers flanking the SSRs that were identified by the module. The TROLL module was configured to find SSRs of mono-, di-, tri- tetra-, and pentanucleotides with lengths of at least six bases.

The list of SSRs produced by the TROLL module was used as input in the Primer3 program, and a list of primer pairs for the amplification of the microsatellite loci was returned. The following input parameters (*e.g*., GC content, TM, and primer length) for the Primer3 software were chosen to fine-tune the process and produce the best results: Product Size Range: 100-400; Max 3' Stability: 250; Primer Size (Min, Opt, Max): 18, 22, 27; Primer Tm (Min, Opt, Max): 45, 60, 68; Max Tm Difference: 1; Primer GC% (Min, Opt, Max): 30, 80; Max Self Complementarity: 6; Max 3' Self Complementarity: 3; Max #N's: 0; Max Poly-X: 4. The other parameters were set at their default values. The program ensures that groups consisting of adjacent SSRs are flanked by only one primer pair, thus accounting for non-exact repeats.

A total of 377 primers were synthesized and used in the genetic analysis. The denomination "EST" was added to the primers' nomenclature to indicate their origin. They were selected based on the presence of perfect and compound motifs containing hexa-, tetra-, tri-, di- and mononucleotide repeats, with a minimum of 36, 49, 18, 12 and 11 repeat lengths, respectively.

### DNA extraction and PCR conditions

Total DNA from the plant materials was extracted from fresh young leaves following the procedure described by [56]. The whole set of markers was initially tested using the same amplification conditions and carried out in a final volume of 15 μl, containing 15 ng of DNA, 0.3 μM of each primer (Forward and Reverse), 0.25 mM of each dNTP, 5% DMSO, 10 μM of Tris-HCl (pH 8.3), 50 mM of KCl, 1.5 mM of MgCl_2 _and one unit of Taq DNA polymerase. The reactions were amplified in a GeneAmp 9700 thermocycler (Applied Biosystems), programmed for one pre-cycle at 94°C for 5 min, followed by 30 cycles at 94°C for 1 min, the specific annealing temperature of the primer for 1 min and 72°C for 1 min, and a final step at 72°C for 7 min. Electrophoresis was conducted with silver stained 6% denaturing polyacrylamide gels [57].

### SSR marker analysis

The 377 primers were screened for amplification quality using the 6 genotypes of *P. vulgaris *available at Embrapa Rice and Beans. Four of these genotypes were parents of two mapping populations: BAT93 X Jalo EEP558 and CNFM7875 X "Laranja". From the primers that showed scorable amplification, those that also produced specific amplification products and amplified consistently across individuals were selected to be used in the transferability analysis and genetic characterization of SSRs. The annealing temperature was adjusted individually for each primer pair.

The set of 107 SSR markers (102 genomic and five EST-derived markers) that were previously developed [2,14,38] were also used in the transferability analysis and genetic characterization of SSRs. These markers reportedly produced specific and robust amplification products when characterized by [29].

An additional set of 44 newly developed SSR markers were tested for amplification quality and polymorphism between the parents of the two mapping populations. These markers were derived from the enriched genomic libraries according to the procedure described by [14] and were used only in the linkage analysis.

### Cross-amplification of EST derived and genomic SSR markers

A total of 167 SSRs that generated clear and specific bands, of which 102 were previously published and 65 were newly synthesized EST-SSRs, were selected for the transferability analysis. The selected set of SSRs were screened for transferability across 11 species, representing four important tribes and one subfamily of the Leguminosae. Two genotypes were taken from each of the species *P. vulgaris, Medicago sativa, P. lunatus, P. coccineus, P. acutifolius, Vigna mungo, Vigna angularis, Vigna unguiculata, G. max, Arachis hypogaea*, which is from the Papilionoideae subfamily, and *Dipteryx alata*, which is a more distantly related species from the Mimosoideae subfamily. PCR was conducted using the conditions described above, and a cross-amplification that produced clear and specific bands, as visualized on polyacrylamide gels, was considered successful. Allele sizes were estimated by comparison with a 10 bp molecular weight ladder.

### SSR genetic characterization

The entire set of 167 SSR markers that were used for the cross-amplification analysis were characterized for the polymorphic information content (PIC) number of alleles, observed heterozygosity (Ho) and gene diversity using PowerMarker [58]. The analysis was assessed in 16 common bean genotypes representing six different and commercially relevant grain classes, representative of the Mesoamerican gene pool (genotypes with small seeds), which included one accession of each of the grain types "Carioca - Carioca bean", "Roxo - Pink bean", and "Rajado - Pinto bean", eight of the "Preto - Black bean", and the Andean (genotypes with medium and large size seeds) represented by two accessions of "Branco - White bean" and three accessions of "Mulatinho/Jalo - Jalo bean".

### Linkage analysis in the BAT93 × Jalo EEP558 population

A total of 315 SSR markers that were not previously mapped in the common bean and produced scorable PCR products (302 new EST-SSR and 13 new genomic SSR markers) were used to screen for polymorphism between the parental lines BAT93 and Jalo EEP558 (BJ). The polymorphic markers were genotyped in a progeny consisting of 74 recombinant inbred lines (RIL) in the F8 generation. An entire set of new segregant markers was integrated into a framework map composed of 123 SSR markers previously mapped in the BJ population [29]. The Chi-square test (χ^2^) was performed to test for deviations of genotypic classes from the expected Mendelian inheritance ratios of 1:1 (p < 0.05) for each marker. The False Discovery Rate (FDR) was determined for the χ^2 ^tests from the observed p-value distribution and used to correct for multiple comparisons by controlling the probability of false positives [59]. The polymorphic SSR markers were integrated in the framework map through a linkage analysis performed using Mapdisto version 1.7b (http://mapdisto.free.fr/). Linked markers were first placed into linkage groups using the "find group" command with a LOD score ranging from 3.0 to 5.0 and a maximum recombination fraction (theta) of 0.30. The recombination fractions were converted into map distances using the Kosambi mapping function. The "order sequence" command was used to identify the most probable marker order within a linkage group, and the "ripple" command was used to return the best order.

### Putative function of mapped EST-SSRs

The functions of the EST sequences containing SSRs that were polymorphic between the parental lines BAT93 and Jalo EEP558 were predicted through similarity searches from GenBank databases (http://www.ncbi.nlm.nih.gov/BLAST) using the BLASTX algorithms. The greater *E*-value was considered to assign a putative function to the EST-SSR markers.

## Authors' contributions

RPVB coordinated the study, participated in analyzing the data and wrote the paper. RAVG and TCOB performed plant genotyping, conducted the genotype polymorphism survey and interpreted results. PNR and CB co-wrote the paper. WSM performed the bioinformatic analyses and primer design. LCM coordinated and supervised the experiment implementation. MSC assisted in the manuscript preparation. All authors read and approved the final manuscript.

## Supplementary Material

Additional File 1 **Table S1. Characteristics of 302 EST-SSRs markers derived from *Phaseolus vulgaris *public databases**. Primer annealing temperature (AT) that produced scorable PCR products. Click here for file

Additional File 2 **Table S2. Cross-amplification of 167 SSR markers on 11 Legume species**. The expected allele size (EAS) for each marker is indicated. Click here for file

Additional File 3 **Figure S1. Transferability of SSRs across species of the *Leguminosae *family**. The electrophoretic pattern at BM98 (Panel A), PVEST260 (Panel B) and PVEST272 (Panel C) revealed on a polyacrylamide gels across species of the *Leguminosae *family: 1- *Phaseolus vulgaris*; 2 - *Medicago sativa*; 3- *P. lunatus; *4- *P. coccineus; *5- *P. acutifolius*; 6- *Vigna mungo*; 7- *V. angularis*; 8- *V. unguiculata*; 9- *Glicine max*; 10- *Arachis hypogaea*; 11- *Dipteryx alata*. Click here for file

Additional File 4 **Figure S2. Segregation of informative SSR loci in *Phaseolus vulgaris***. Polyacrylamide gel resolution and detection by silver staining of SSR PVEST272 (Panel A), PVEST279 (Panel B) and PVEST336 (Panel C) in BAT93 × Jalo EEP558 population. Lane 1 is a 100 *bp *ladder size standard with the sizes of some fragments indicated in base pairs; lanes 2 and 3 are the two parents, indicated by the arrows, followed by the segregant population. Click here for file
