# Supplementary material for: The characterization of a new set of EST-derived simple sequence repeat (SSR) markers as a resource for the genetic analysis of Phaseolus vulgaris
Source: BMC Genet. 2011 May 9;12:41. doi: 10.1186/1471-2156-12-41 (PMC3102039; doi:10.1186/1471-2156-12-41)
Supplement: Additional File 1 — Table S1. Characteristics of 302 EST-SSRs markers derived from Phaseolus vulgaris public databases. Primer annealing temperature (AT) that produced scorable PCR products. [file 1471-2156-12-41-S1.DOC]

**Table S1. Characteristics of 302 EST-SSRs markers derived from *Phaseolus vulgaris* public databases. Primer annealing temperature (AT) that produced scorable PCR products.**

| **Locus** | **GenBank accession** | **Microsatellite motif** | **Product size** | **FWD primer** | **REV primer** | **AT (^o^C)** |
| --- | --- | --- | --- | --- | --- | --- |
| PVESTBR001 | ALV_001_B03_G1 | (AG)21 | 299 | TGTTTGGTTAACAATTCGATGC | TTTCAAGCGGGAAAAGAAATAA | 56 |
| PVESTBR002 | ALV_001_C01_G2 | (GCT)6 | 253 | ACCACCAGAAAATGGAACAACT | ATTAGTAGATTCAGCCACCGGA | 56 |
| PVESTBR003 | ALV_002_G04_G2 | (CTC)7 | 279 | AGAACCTCAAGAGGGGTTTCTC | AGGCCATTGTGTTCTTCCTAAA | 56 |
| PVESTBR004 | ALV_003_H03_G1 | (CAG)7 | 274 | TGGATTCGAGACATGAGATGAG | GATTCGGTTCATCCTCTCCAG | 56 |
| PVESTBR005 | ALV_004_B02_G1 | (GT)6 | 114 | GAAGAAATCGTCGTTGTTGTGA | CAATAGAGATCGTGGGAAGCAT | 56 |
| PVESTBR006 | ALV_004_B03_G1 | (AG)35 (G)8 | 151 | TTTTGAGGATTGGGAATATTGG | TCAAATGGACTACGATTAACTTGC | 56 |
| PVESTBR007 | ALV_006_B01_B2 | (T)6 (TA)15 | 230 | TTCGTCTGATTCTATTGGAAGAAA | ATGGGGTTGAACTCATCCTAGA | 56 |
| PVESTBR008 | ALV_007_B10_G1 | (AG)17 | 188 | ATGATGAGGAAATATTGGTGGC | GTGAAGGGGAAGAGAAAAGGTT | 56 |
| PVESTBR009 | ALV_007_C06_G1 | (CA)12 (TA)15 (T)8 | 365 | GATTTGCTTGATCACATTCCCT | GAGGTGCCAAAGTATCACATCA | 56 |
| PVESTBR010 | ALV_007_C08_G1_1 | (CT)11 (A)6 (A)6 | 269 | GTAGGTATCGGTGGAACTCCTG | GAAGCTGCAAACAATACTGCTG | 56 |
| PVESTBR011 | ALV_007_C11_G1 | (GCA)6 | 398 | CTGCTGAGATTTTATCGAGGCT | CTGCTGTGTTTGTTGGTTCAAT | 56 |
| PVESTBR013 | ALV_008_E08_G1 | (CT)13 | 192 | CAACGTCCATAGGTTTCATTCC | GATGAAGAGCCTAGTGATTGGG | 56 |
| PVESTBR015 | ALV_009B_C02_B1 | (A)6 (T)7 (T)6 (T)6 | 239 | TTTCTTCTTCACACTCCCCATT | GACATGGGAACAGCAAAATGTA | 56 |
| PVESTBR016 | ALV_009C_G08_B1 | (TA)7 (T)6 (T)7 | 321 | CAACAATAACCCCGTTTTGAAT | GGAGGAGAAGGAGAAGGAGAAG | 56 |
| PVESTBR017 | ALV_009C_G08_B1_1 | (TCT)9 (T)8 | 198 | TTCTCCTTCTCCTTCTCCTCCT | CGGAATACCCTTTCACTTCTTG | 56 |
| PVESTBR019 | ALV_010C_D06_B1 | (G)10 (C)6 (A)10 (G)9 | 144 | AATAGAAGCAAATGCCACGAAC | CTTTGTTCCCCTTTATAACGCC | 56 |
| PVESTBR021 | ALV_010D_E08_B1 | (A)7 (A)6 (A)6 (A)8 | 173 | GCACTGCTAGCGAGATTAGACA | AACACCAGCTTTGTCTTCTTCC | 56 |
| PVESTBR022 | ALV_010D_G06_B1 | (T)7 (T)8 (A)6 (T)7 | 257 | CAGAAATTCAAGGAAAGGTTGG | TGTGCTATTCTTGTTCCATTGC | 56 |
| PVESTBR023 | ALV_011B_F10_B1 | (T)7 (T)7 (A)6 (T)6 (T)6 | 361 | CAGTCGATCAATTCAGTGGAGA | ACAAAACACCTCCACAGTCAAA | 56 |
| PVESTBR024 | ALV_011D_A05_B1 | (GAA)6 | 281 | ATGTCAGCTCGATTGATGAAGA | GATTTGGGATTTGATACGGAAA | 56 |
| PVESTBR025 | ALV_012C_B11_B1 | (TC)8 (G)11 | 226 | CTTTCTCTTCACTTCTCATTGGC | GATGCCGTTGTTGTCAGAAAT | 56 |
| PVESTBR026 | ALV_012C_E03_B1 | (TC)10 (T)7 | 343 | TTACCCCACAATCCTGCTATCT | AGATAGGAACTTGGGAAGGAGG | 56 |
| PVESTBR029 | ALV_013C_A09_B1_1 | (T)7 (TA)6 | 143 | CCCAAATTATCCTCATCCAAAA | AAAGCAAATGGTAATTGGTTCC | 56 |
| PVESTBR030 | ALV_013C_B12_B1 | (TA)6 | 188 | GAGGAAACCAGAAATGCTTGAC | GTTTTATGACAAAGATTTTGCTGC | 56 |
| PVESTBR032 | ALV_013C_C07_B1 | (TGG)6 | 151 | GGGAGGAGGTGACTTGTCTTTT | ATCACCATAACCACGATCCCTA | 56 |
| PVESTBR033 | ALV_013D_A12_B1_1 | (TA)7 (A)6 (T)10 | 311 | GTACGGGGATTATGTGCAATTT | CTCAGCAGGCCTATCTCTCAAC | 56 |
| PVESTBR034 | ALV_013D_C01_B1 | (AC)6 (T)6 | 196 | AACTTCAATCTGGGGCTTAACA | CCAGATAATGCTGTGCCTATGA | 56 |
| PVESTBR035 | ALV_013D_C01_B1_1 | (T)7 (A)104 | 192 | GACTCAAGTTTATCCATTTGCT | CGGAGGAATTTTAAAGGG | 56 |
| PVESTBR036 | ALV_013D_D02_B1 | (T)8 (G)9 (C)6 (A)10  (A)7 (T)6 (A)6 (T)6 | 318 | AAAACGGTACCTTGGGAT | AAAACCTTTCAAAATTGGGT | 56 |
| PVESTBR039 | ALV_014A_D02_B1_1 | (A)61 (G)6 (T)6 | 337 | TGGCATATGAAAGATTTGTTGC | AAGCTAGCAGTTTTCCCCG | 56 |
| PVESTBR040 | ALV_014B_C08_B1_1 | (T)7 (T)8 (T)7 (T)8 | 377 | GCAAGAATGTGCTCAGAGAGTTT | CACCAGACCTGTTTTGTCACAT | 56 |
| PVESTBR041 | ALV_014B_D11_B1_1 | (A)6 (A)79 (G)8 | 297 | TGGGTTCTGTACAACATTCTGC | AGGGGTAGGGGTAAACTTGG | 56 |
| PVESTBR042 | ALV_014C_G08_B1 | (AG)14 | 177 | CGTGTTGGAGAGAGAGTG | GTTCCAAAGGGATTATTACTG | 56 |
| PVESTBR043 | ALV_014C_H09_B1_2 | (T)7 (T)6 (A)6 (A)7 | 217 | GTTGCTCCTCATGCAAACACTA | AGGGATTGCCTGCAGTTTAATA | 56 |
| PVESTBR044 | ALV_014D_D08_B1 | (A)7 (T)7 (T)6 (A)7 | 316 | GGACAGATTGGAGAAAAGCATC | CTACCACAACCTCAACAAACCA | 56 |
| PVESTBR045 | ALV_014D_H03_B1 | (A)6 (GAA)6 | 125 | GCTTATACGTCACCGTTTTGGT | CTTCTCTCTGTTTTCGCTCTGC | 56 |
| PVESTBR046 | ALV_014D_H11_B1_1 | (A)6 (A)6 (A)6 (A)6 | 299 | CAAACACTTCAGTTGACATGGG | GTTATCACTGTCCCTGCTACCC | 60 |
| PVESTBR049 | ALV_015A_G09_B1 | (CT)7 | 302 | CGTCCGTACTTTTCAAACTCAA | TTGTCAGTATCCGAGGAAAGGT | 56 |
| PVESTBR050 | ALV_015B_A06_B1 | (AGC)6 | 372 | CGTCCGCTTTTCTTTTAAGTTG | CTTCGGAGTTTGCTTTGGAG | 56 |
| PVESTBR052 | ALV_015D_B11_B1 | (A)10 (G)7 (A)12 (G)8 (A)6 (G)9 (A)10 (A)7 (G)6 (G)9 (T)6 | 256 | GGAAGGGAGGGGAAAAGTT | TTTTCTTCACCCCAAACCA | 56 |
| PVESTBR053 | ALV_015D_D09_B1 | (GAA)7 (CAA)6 | 343 | TCGAAGAGGAAGAAGAGGAGGT | CAGTCCTCGATCTCTCCGTACT | 56 |
| PVESTBR055 | ALV_016B_C02_B1 | (TC)12 (TC)17 | 308 | TTTTATGGTGGAACAAGCTCCT | GCCATGTCTTGGAAAATCTCTC | 56 |
| PVESTBR056 | ALV_016B_D05_B1 | (GT)6 | 333 | CTCGAGAGGGGAAAGAGAGA | TGATTAAGGCGACTTGGTTACA | 56 |
| PVESTBR057 | ALV_016B_D05_B1_1 | (GA)9 (A)11 (A)6 (T)6 | 318 | TAGCAAATGCCAAATGGTAGTG | GGAAATACCCAACAGGAATCAA | 56 |
| PVESTBR058 | ALV_016C_E03_B1_1 | (T)7 (A)59 | 263 | GAGGCAGGGAAGAACCCTCTAT | CGTAAGCTTGGGCCCCTC | 56 |
| PVESTBR059 | ALV_016D_A04_B1 | (TC)7 | 230 | CCCAGGGAGAGTGACGTTG | TGATCGTAGATGTGGATGGATG | 56 |
| PVESTBR061 | ALV_017A_E11_B1_1 | (ACA)6 | 137 | TGTTGCCAGTCTTCTCACAAGT | CTTTATCAATGCCTGAACCTCC | 56 |
| PVESTBR062 | ALV_017B_G05_B1 | (TCA)10 | 118 | ATGTCAAAACTTCCCAGTTGCT | AGTGAAAGAGTGGGTGTGTGTG | 56 |
| PVESTBR063 | ALV_038_C08_G1 | (GA)6 | 209 | GGAAGAAGAGGAAACACTGCAT | TCTCAAGGGCTTTAACTTCAGC | 56 |
| PVESTBR064 | Contig1043.seq | (AG)6 (T)6 | 400 | CTTCAATTGCCAGATTCATTCA | CAAAGGGATGATTTTCTTCAGG | 56 |
| PVESTBR065 | Contig1077.seq | (CTT)7 | 315 | TCCTCCTCCTCTTACAGTTTGC | GGTAGCTGGCTTCTCAAAGAAA | 56 |
| PVESTBR066 | Contig1080.seq | (TCT)7 | 382 | GTACTTTTCTGGGTTCAACGCT | GATTTTGGATTGTTCATCGTCA | 56 |
| PVESTBR067 | Contig1084.seq | (CT)8 | 240 | CACTGAGGTTTTCCTCTGCTTT | TGTTGAAGGCAAAACATGAAAC | 56 |
| PVESTBR068 | Contig1087.seq_1 | (T)6 (T)6 (T)7 (T)6 | 368 | CAATTCCACAAACACTCCTCAA | GTTACATAAGCACCCCAAGCTC | 56 |
| PVESTBR069 | Contig1094.seq | (AG)7 | 147 | AGAGAGGAAAACAGAAACAAACG | GATTTGACGGTAATCGGAAAAG | 56 |
| PVESTBR070 | Contig127.seq | (AG)8 | 278 | GTGTCGGTTGTGAGACAGGAG | TGTAAACTGAACTGCAGAGGGA | 56 |
| PVESTBR071 | Contig1282.seq | (TC)8 (CTT)6 | 112 | TCCTCTCTCTCTCATTTCCAATC | AGCCATTATAGTGCGTTTTGGT | 56 |
| PVESTBR072 | Contig1299.seq | (AT)11 (AT)6 (AT)7 (TA)10 | 219 | CATGACTAGACAACGCCATCAT | GGTTGTAGAATTGGATTCCTGC | 56 |
| PVESTBR073 | Contig1338.seq_2 | (AG)6 | 150 | TGGAACAAACACCAAGAACAAG | GAATCATGTGAAGTGCCGC | 56 |
| PVESTBR074 | Contig1360.seq | (GTG)6 | 317 | GCTTTTGTTGGAGGAGAATGAG | TCTTCAACCCATGTTGAGAATG | 56 |
| PVESTBR075 | Contig138.seq_1 | (TA)10 | 368 | GATTACCCTCGCACTCTCACTC | TCTAATGCTATCCATGGTCCCT | 56 |
| PVESTBR076 | Contig1383.seq_1 | (TA)11 | 128 | CTGCGTGTAATTGAACGTGATT | GAAGAGGACATGATTAGGCACC | 56 |
| PVESTBR077 | Contig1414.seq | (ATA)8 | 334 | TTTTGTTTGTAAACCCAAGGCT | TTGCACTTACACTTTATGGGAAAA | 56 |
| PVESTBR078 | Contig1445.seq | (CAC)7 | 290 | ACATATTTCAGATGTGGAGGGG | TTGAGGATGTTGAGTTGGTCAC | 56 |
| PVESTBR079 | Contig1519.seq_3 | (TC)7 | 398 | TGATAGTTCTTCCTGTTGCCCT | ATTCGTATGCTAAATTGGGTCG | 56 |
| PVESTBR080 | Contig1564.seq_1 | (A)35 (A)11 (A)6 | 258 | TCTTGACACCTTTTCACCCTTT | CCGCCCCTGCTTTATTTT | 56 |
| PVESTBR081 | Contig1573.seq | (T)6 (A)6 (A)7 (T)8 | 366 | TCAGTGTTTAGGGTCTCCATGA | CATGAGGACACAACTGCAGAAT | 56 |
| PVESTBR082 | Contig1599.seq | (AG)9 | 348 | ATGTTGTTCTTCGAGCAGTTCA | ATACAAGTTACAGGGTGTGGGG | 56 |
| PVESTBR083 | Contig1600.seq | (TC)6 (TC)6 | 173 | TCCATACAAATTTCACACTCGC | CGGAGTGTTTGGTACGGATATT | 56 |
| PVESTBR084 | Contig1609.seq | (T)6 (T)7 (ATA)6 | 309 | ATTAATCGGCTCGGTCAAAGTA | GAATTCACCATGGAAAGTGATAAA | 56 |
| PVESTBR085 | Contig161.seq_1 | (TC)6 | 224 | TCCCAAATCCCACTATATTTGC | TTCTCTGTTACGCATGAGCCTA | 56 |
| PVESTBR086 | Contig1621.seq | (CGA)7 | 147 | TCTCTCTTAACCGTCAGAACCC | TGAAATCTTCGTGTTCGTTTTC | 56 |
| PVESTBR087 | Contig1667.seq_1 | (AG)8 | 354 | GGACTTTCAATCCTCCTGAGTG | TTCAAAGCTCCTGAAAGAAAATG | 56 |
| PVESTBR088 | Contig1671.seq | (T)6 (T)7 (T)6 (A)6 | 145 | GCCGAGTTTTGAGTACAGTTGA | TTTACCAATTCTCCCATCCATC | 56 |
| PVESTBR089 | Contig1692.seq | (AT)7 (T)7 | 354 | ATTGTGTGTTTTGCTGTTGGTC | TTTGACAATCATGCTTTTGCTT | 56 |
| PVESTBR090 | Contig1713.seq | (AGA)7 (A)13 | 301 | CTAACGACACTGCTGGAAACAG | CATATCAGCACTTTTGGCAGAC | 56 |
| PVESTBR095 | Contig184.seq | (AG)12 | 194 | CTTTGGTCGAAGAAGCAGAGAG | GACCTTAGCGAGAGGTTGAGAA | 56 |
| PVESTBR096 | Contig186.seq | (A)6 (A)51 | 164 | ATCAGCTCTCATTTGCATCTTT | GAAAATTTAGAGGGGCCG | 56 |
| PVESTBR097 | Contig1877.seq | (TG)6 | 135 | TGCCATTTCTTCTCTTTGTGTG | CATCGAGACCTTCAGTGACTTG | 56 |
| PVESTBR098 | Contig1880.seq | (CTT)7 | 134 | TCTTTAACAGCGCACACACTTT | GTTGGAAACGACAGTAGGAACC | 56 |
| PVESTBR099 | Contig1916.seq | (CTT)7 | 168 | TCATCAATGGCTACCTTTCCTT | GTTTTGGCCTAGTGGTTTTCTG | 56 |
| PVESTBR101 | Contig1984.seq | (T)6 (AT)7 (A)6 | 296 | TTCTTCTCCTTTCGATGTGTGA | TAACAAAAGCACGCCCATATC | 56 |
| PVESTBR102 | Contig2031.seq | (ATGAAC)6 | 298 | ATCCAGATGAGTGGGTTTCAGT | TCGTACACCTCCTCGAATACCT | 56 |
| PVESTBR103 | Contig2051.seq | (AG)8 | 395 | GGTGGGAGATGACAGAAGAAGT | TCTATGGATAGTTCGGCAAGGT | 56 |
| PVESTBR106 | Contig2172.seq | (T)6 (AAT)8 (T)8 | 338 | GTGAGGGTTCTGTGGATTCTTC | ATTGACACAACAGCCAATATGC | 56 |
| PVESTBR107 | Contig2184.seq | (AG)8 | 391 | TTCTCAGTGCGCGCGTGT | CCATCATCTGCGTGGTCTTGTT | 56 |
| PVESTBR108 | Contig2220.seq | (GA)9 | 309 | CTTCGTTGGTTTCTCTTTTGCT | TGCATTATCTGAATCTCCCTCC | 56 |
| PVESTBR109 | Contig2222.seq_1 | (AAG)6 | 309 | GGAAGAAGAAGGAACTTGCAGA | CGGTAGATGTATCCTCCTCCAC | 56 |
| PVESTBR110 | Contig2223.seq | (G)11 | 387 | CAGTTTGCTTGATTGGACTCAG | CCCAACACTGATATAGCGTCAA | 56 |
| PVESTBR111 | Contig2302.seq_1 | (CAG)6 | 103 | CATAACAACAATGAAGGAGGCA | TTCATCATTTCTTCGTTTGTGG | 56 |
| PVESTBR112 | Contig2339.seq | (CTT)7 | 318 | CCTTCATCGTGTAATTCCTTTGT | GTCGGTGTCTACAACATCCTCA | 56 |
| PVESTBR113 | Contig24.seq | (AT)6 | 292 | GCTAGCTTCAAAATACAAAAGGCT | CTTTCTCCCCTATCTTGGCTTT | 56 |
| PVESTBR114 | Contig2442.seq | (TC)7 | 143 | TTTCCCCTCCTTTCTCTTTTGT | CTTCGTACGGGGTTTTATTCAG | 56 |
| PVESTBR115 | Contig2442.seq_1 | (GA)9 | 334 | CGTGACCACATCTCTCTGAAAA | GGAGAGGAGGAAGAGGAAGAAG | 56 |
| PVESTBR116 | Contig2456.seq | (ACC)6 | 381 | CACTCACAATTCAACTTTCCCA | GTTGATCTTCACGCTCCAGTC | 56 |
| PVESTBR118 | Contig2656.seq_1 | (T)6 (T)6 (A)8 (G)6 (A)8 | 320 | AAGAGAAATGGAGCTGTTTTCG | TCATCATTCAAACGATTCAACA | 56 |
| PVESTBR119 | Contig2680.seq_4 | (T)6 (A)9 (A)7 (A)6 | 344 | ATTGCCATGCTTAGTGTGTGAC | TTTTCTGAGATGACCCATAGACC | 56 |
| PVESTBR120 | Contig2757.seq_1 | (CT)6 (C)6 | 229 | CTGAAGCCATGGATGTACTGAG | AAAGTACCGGAGAAAACGACAA | 56 |
| PVESTBR121 | Contig2769.seq | (ATG)6 (T)7 | 340 | CAATTTATTCAGGAAGCCTTGG | GCATATGAACATGTCTGGTTGG | 56 |
| PVESTBR122 | Contig2805.seq | (GA)6 | 336 | GCACTTGCTCTCATACCTATACTTTG | TGTCAACTTCTGTGGAGTGCTT | 56 |
| PVESTBR123 | Contig2809.seq_1 | (A)6 (A)7 (A)6 (T)6 | 360 | GTTTTAGCAAAAGCCAAACACC | CACAATTTCCCAGCTTCCTAAC | 56 |
| PVESTBR125 | Contig2846.seq | (TA)8 | 320 | CATGGAGAAGAGCACAAACAAG | AATAACCAACCACACACCACAA | 56 |
| PVESTBR126 | Contig2888.seq_2 | (CCA)6 | 391 | CCTTACGTTCCTAAACCACCAG | GAGCAATGGGGATTATGATGTT | 56 |
| PVESTBR127 | Contig2889.seq | (T)6 (A)6 (T)7 (A)6 (A)6 (A)8 (A)6 (A)11 | 292 | CGTGCTCAAAGACGTAAAACAG | CAAAGATAATGCTATCCCAGGC | 56 |
| PVESTBR129 | Contig2926.seq | (GT)6 (T)8 | 198 | TTCCTGGCCTTAACACAAAGAT | GAAAGTCGCATAGGAGAAACCA | 56 |
| PVESTBR130 | Contig2927.seq_1 | (AC)6 | 152 | CGTCGTGTCTCTCTTTGTGTTC | AAAACAGGTCCCTCACGATTTA | 56 |
| PVESTBR131 | Contig2937.seq | (CAG)7 | 259 | CTCTTCTCTGCTCTCACGAGGT | GGGTGAACATAGGGCATGTAAT | 56 |
| PVESTBR132 | Contig2965.seq | (AAG)6 | 284 | AAGAGAAGCCTCAGGAAGAGGT | CTTTTCCTCACTTTCCACAACC | 56 |
| PVESTBR134 | Contig2970.seq | (TC)6 | 165 | CTCAGAGGAGAAGAAGATCCCA | GGTCATTCTCATCCACCAAAAT | 56 |
| PVESTBR137 | Contig306.seq | (AT)15 | 185 | TCATTCAGTGTGCAAATAACCA | ACTACAGCAACAGCCACAGAAA | 56 |
| PVESTBR138 | Contig3062.seq | (GCT)6 | 341 | CATTTCTCAGCAACTCCTTCCT | ATAACAGTATCCCAATCGGTGG | 56 |
| PVESTBR140 | Contig3077.seq_3 | (A)7 (A)7 (A)7 (T)6 (T)7 | 340 | AGCCCCTCACCAATAGACTACC | TGGATCGTTAGATAGGAATCCG | 56 |
| PVESTBR141 | Contig3101.seq_1 | (AAT)7 | 212 | AGTTTGAGAGAGAGATGCAGGG | AGACAAGTGAATTGCGGATTTT | 56 |
| PVESTBR144 | Contig3126.seq_1 | (GAG)6 (T)13 | 383 | CAGCCACTGTCAAACAAGAAAG | CTCCTCAACACCAAATCAGACA | 56 |
| PVESTBR145 | Contig3166.seq | (TCT)6 | 226 | CTTTAACAGCGCACACACTTTC | AAGCATACAGAGGAAGCTTTGC | 56 |
| PVESTBR146 | Contig3205.seq | (GA)9 | 320 | CTCAGGATTCAGAGAGAAA | ATAATCACTTCACTGTCTTGTC | 56 |
| PVESTBR147 | Contig3274.seq | (TA)9 (ATA)9 | 259 | TAGAAACACGGTTTGCTTGATG | TCTTGCAAAGAAACCATACGCT | 56 |
| PVESTBR148 | Contig359.seq | (A)19 (TC)6 | 320 | TGATGTCTATGCTGGAGGTACG | CTGCAAAAGAGTGAGTCCCTG | 56 |
| PVESTBR150 | Contig437.seq | (TA)7 (A)7 | 183 | TGACACAGCTAAAAGTTGTCCTTC | TGCTGCAAAATGTCTTAATTGC | 56 |
| PVESTBR151 | Contig451.seq | (TCA)7 | 211 | GGCAATGTCATGATTGAGAAGA | ACGCTGTTTGAAGCCATTATTT | 56 |
| PVESTBR152 | Contig452.seq | (A)6 (GAG)8 | 351 | ATTTCATTCCCTCACACAATCC | GCACACTAGCAGGAACACTCTG | 56 |
| PVESTBR157 | Contig548.seq | (TC)8 (A)6 | 385 | GCGACCTACTCAGTCTTTTGCT | GAAACTTACACACTGCCTGTGC | 56 |
| PVESTBR158 | Contig551.seq | (TA)9 | 107 | ATCTCGACAAGGCATACATAGC | GCCATCAACATCTAAGAGCAAA | 56 |
| PVESTBR161 | Contig691.seq | (TA)6 | 252 | TCATCATCACCTTGCACTCTTC | CTCACTGCTCAACAACACAACA | 56 |
| PVESTBR164 | Contig771.seq_2 | (AT)6 | 315 | CCCTCAAGAATTGATCCTCATC | CATTAATCCTCCCCAGCAAATA | 56 |
| PVESTBR166 | Contig802.seq | (CT)11 | 136 | AAAAGAGTTCCGCGTCAGTAAG | GAACATATTCACCAAAAGATTTGC | 56 |
| PVESTBR168 | Contig838.seq | (TC)9 | 153 | AGAGCCGTCACTTGTTTGTTTT | GTATGTGAAGCCCCTGAAACTC | 56 |
| PVESTBR169 | Contig845.seq | (TG)7 | 240 | CACACACCAAACCAATCACTCT | AGGTCGTAGAGAATGACGCTGT | 56 |
| PVESTBR172 | Contig882.seq | (TTC)6 | 163 | AAATAATCAGCGACGACGAAAT | CCACTCGATAACCTTCTTTTGG | 56 |
| PVESTBR174 | Contig956.seq | (ATG)6 | 266 | ATTGGCAGAAGGAACAGAGAAG | TCACTAGAAGGGGTTGCAAAAT | 56 |
| PVESTBR175 | LVS_005_H01_b2 | (AT)6 | 354 | AGGAGGAAAGATGTGAAGACCA | TCACCAACAAACTTCATGATCC | 56 |
| PVESTBR176 | LVS_010_H04_b2_1 | (TA)22 | 392 | CTAAAGGAGTTTGTGCGATGTG | ATCCGACTCATAAAAGGGAGGT | 56 |
| PVESTBR178 | LVS_025_B10_b2 | (AG)7 | 118 | AGATGTTTAGAGAGAGGGTGGC | AAACTCAACCATCACCACCAC | 56 |
| PVESTBR179 | LVS_028_B03_b1 | (GA)7 | 330 | GACGGAGCTACTTCGGCTG | AAAAGGACTTTGGCTTTGTGAA | 56 |
| PVESTBR180 | LVS_028_F02_b1 | (TTC)7 | 177 | CAGTGAAACCCTTCTTCGTACC | CAACTTTCAGACCAAACCTTCC | 56 |
| PVESTBR184 | LVS_035_A06_b1 | (TTA)6 | 315 | GCAAAACTAATGCCGAATAAGG | TTCCCGATCTGAACTTACCACT | 56 |
| PVESTBR186 | LVS_040_F02_b1 | (AAG)6 | 208 | GGAAACCCAACTGAGAGAG | GAAGAAGAAGAATGAGAAGCAA | 56 |
| PVESTBR189 | LVS_042_D06_b1_1 | (GCA)8 | 219 | GATTTGTTGAAGCCAAAGGAAC | TACAAATTCCGTTCGATCACTG | 48 |
| PVESTBR192 | LVS_045_H02_b1_1 | (CT)6 | 257 | ATGATTGTAGCAGCTGGAGGAT | AAGGGAAGTGCAGTGAAATGTT | 56 |
| PVESTBR193 | LVS_048_D03_b1 | (TG)6 (T)6 | 324 | GTGGAGGGTTATGTTGCTTCTC | TTCCATTGAAAGTGGTCTAGTAATTC | 56 |
| PVESTBR194 | NOD_203_D08_063 | (TA)8 | 292 | ACTTAGGTTAGGCCTTCATCCC | ACCGTCCCATTATTGAACTAGC | 56 |
| PVESTBR195 | NOD_203_F02_016_1 | (CAA)6 | 194 | CACTCTCTCATCTGCGCAATAC | AGGTTCGAATAGCTTCAGCAAG | 56 |
| PVESTBR196 | NOD_203_F12_096 | (GAC)6 (TA)7 | 318 | GAAGAAAGTGTTTTCAATGGGC | GGATAACAAACCCAGTGGCTAA | 56 |
| PVESTBR197 | NOD_211_C08_065 | (TC)9 | 106 | GACGCTAACCGCCACCAC | TGAAACAGCCCCAGAAATACAA | 56 |
| PVESTBR198 | NOD_217_A08_053 | (CCG)8 | 273 | AGAGGGAGGCTCTCAAACTTCT | CTTTATCCGCGTGGAACATT | 56 |
| PVESTBR199 | NOD_217_D08_062 | (GCG)7 | 336 | AAAGACGATGCAGTTGGAAAGT | GTTATTGTGTTCTCATCCGCAA | 56 |
| PVESTBR200 | NOD_217_H04_032 | (TC)6 | 128 | ACAGCTGGAGACTGAGTTACCG | ATGAGAAGAGATTGGAAGCGAG | 56 |
| PVESTBR201 | NOD_218_A09_066 | (TCT)6 | 379 | TCACCTCACCAACTAAACCACA | AGAGAGAGCAAAAGGCACATTC | 56 |
| PVESTBR202 | NOD_218_H07_061 | (A)6 (GA)7 (T)6 | 239 | TCTCTAGAAACCACCTTCGCTC | CGATTGTTGCATCCTAAATCAA | 56 |
| PVESTBR203 | NOD_220_G09_069 | (TGC)6 | 304 | GGTGATCAGAAGAAGGGTATGC | CACATCTCAAAACAAGAGCACC | 56 |
| PVESTBR204 | NOD_223_E06_040 | (AG)13 | 111 | AAGCGGTAGTTGAAATTTTGGA | TCGACGGTTATGCTAATCCTTT | 56 |
| PVESTBR205 | NOD_225_C03_019 | (AC)8 | 114 | GCAACAACAATTTGCTTCAAAA | CGTCGTCGTTCATAGTGATGAT | 56 |
| PVESTBR206 | NOD_227_G11_085 | (TA)6 (TA)6 (TA)6 | 250 | TTTGAGTCTCTACACCAACCCA | CCATCTTTCTATACAATTGCACG | 56 |
| PVESTBR207 | NOD_230_E11_084 | (CAA)6 | 334 | AACCTCGTCATCATCACCATC | TAAGAACCGGAAGACGAAACAT | 56 |
| PVESTBR208 | NOD_236_F05_044_1 | (CT)6 | 144 | GATTTTATTGGACCCTCTTCCC | TAACCGTGAGAGACACTTGCAT | 56 |
| PVESTBR209 | NOD_237_H07_061 | (CTT)7 | 230 | TACCTTCTTACTTCCATTCCCA | AGATTGCAGATGGTGAATCTGT | 56 |
| PVESTBR210 | NOD_244_B08_061 | (A)6 (T)7 (T)7 (T)6 | 369 | TCGGATTGCAAGGTAAATTTCT | GACAATGACGAAGACCATTGAA | 56 |
| PVESTBR211 | NOD_244_B08_061_1 | (A)10 (T)6 (T)6 (T)7 | 316 | TTCAATGGTCTTCGTCATTGTC | TTCACACAAGCATAACCATTCC | 56 |
| PVESTBR212 | NOD_245_A12_085 | (CAC)7 | 396 | TTGTTCCAATGAGCATGTTAGG | TCACTCCCTTATGCTGATGATG | 56 |
| PVESTBR213 | NOD_249_C05_034 | (CTT)6 | 319 | CTTTACAAACCCTAACCCGTTG | GTTTCTGCTGACCGTTTTCTCT | 56 |
| PVESTBR214 | POD_003_H05_046_1 | (TA)6 | 235 | TCACCAGAGCTACTTTATGCCA | CAACAAAGACCTTGCCACAATA | 56 |
| PVESTBR216 | POD_009_E05_035 | (CT)7 (TCC)7 | 291 | TTGAGGACTGAGGAGGGTTTTA | AGAGGGAATCTCAGTAGGTCCC | 56 |
| PVESTBR217 | POD_012_A11_081 | (T)7 (AT)6 (T)6 | 248 | CCACCTGATCTTGATTCCTTGT | TCATCATCAGCTAACACCTACGA | 56 |
| PVESTBR218 | POD_014_G03_020 | (CCT)6 | 315 | CCTAACAACTGGTGGAACATCA | CCCTTCTTGCTTAACATCAACC | 56 |
| PVESTBR219 | POD_015_F01_011 | (A)6 (T)12 (A)6 (T)12 | 388 | ATCACGCCCAACTGTCTTCTAT | CAAAACAGCTTGCTATCACTTTCT | 56 |
| PVESTBR220 | POD_018_C08_054 | (AG)8 | 386 | GTTTGGGAAGTGATTGTTACT | TGAAGATGTGGTTTTGATACTC | 56 |
| PVESTBR221 | POD_020_D07_058 | (T)6 (T)8 (T)7 (A)6 | 304 | TTGCTAGTTCTTTCCTCCTTGC | CTGCTACTGCTGCTTCAACTGT | 56 |
| PVESTBR222 | POD_020_F09_075 | (TGA)6 | 231 | GATGGTGGTGGTGATGTTGTT | TTTGCAAGTTCCAGTTTTGAGA | 56 |
| PVESTBR223 | POD_022_H01_012 | (TCT)10 | 206 | CACTTCTTCTTCTTCTTCAT | GACTCTTCTTTCTGACTTTCT | 56 |
| PVESTBR224 | POD_026_B08_061_1 | (GGA)6 | 185 | TTCTTGCTCTCCAGTGTTCAAA | ACCAGGTTTCAGAGACAAGCTC | 56 |
| PVESTBR225 | POD_029_D09_074 | (AT)15 | 312 | ACGACCGTACCTTCAAGACATT | TAACCAAAGCGAAACAAAGTCA | 56 |
| PVESTBR226 | POD_031_B06_045 | (AAG)9 | 248 | CACAACCCTAAACTGGGACATT | GACACCTTCATGATTGGAGACA | 56 |
| PVESTBR227 | POD_036_B08_061_1 | (T)8 (TG)7 | 178 | AGTAGCCAACCTCTACTCACGC | CAACAAATGAAAACAGTATCAACCA | 56 |
| PVESTBR228 | POD_039_G05_036 | (GA)7 | 289 | CACCTTCTGCCTTATCCTCAGA | AGCAGCACTTACCTCTTATCCG | 56 |
| PVESTBR229 | POD_039_G09_068_1 | (GA)8 | 229 | CTTCATCGGTTCCTATCAGAAGA | AGGATGTGCTTGAAGAAGAAGG | 56 |
| PVESTBR230 | POD_040_B03_025 | (AT)15 | 255 | CCATGATGAAAATCCCTTTGTT | AATCCCTAGGATGTAAACGCAA | 56 |
| PVESTBR231 | POD_040_G08_056 | (GT)6 | 330 | TTTCTGGGAGATCATGGAGATT | TACACCTGCCACTCATTGATTC | 56 |
| PVESTBR232 | PVEPSE2003D01.seq | (TCT)6 | 146 | CCACCACCTTCCATTCTGTTAT | ACTCACAGGCCAATACAACCTT | 56 |
| PVESTBR233 | PVEPSE2009C06.seq | (T)6 (TC)6 | 155 | TCTGAAACCTCAAGTTCTAACACAA | CAGATTCATTCTTCTGGTGCTG | 56 |
| PVESTBR234 | PVEPSE2013B07.seq | (C)7 (CT)9 (C)6 (A)6 | 396 | CTACCCTTCCACCTCCTCTTTT | CACCTCTTCCTTTCCTTGAAGA | 56 |
| PVESTBR235 | PVEPSE2014C04.seq | (AT)6 | 198 | AGTTGGAGCAATTTGGATGAGT | ATTTATTGAATGCTAGGCCCTG | 56 |
| PVESTBR236 | PVEPSE2014F08.seq | (TC)7 | 316 | AGATTGCACTGATCCCTTTCTC | TCAGCCTGTTCATAAGCTGGTA | 56 |
| PVESTBR237 | PVEPSE2016A02.seq | (TC)6 | 397 | GCAAGAAAGAAAAGTTCCCACA | CAGACACAGGTTCGAGTTTCAC | 56 |
| PVESTBR238 | PVEPSE2021C05.seq | (CTT)6 | 352 | CTACTCCATAAATTCTCCTTTTGTTT | AAGTCTCCATCGACACATTCTC | 56 |
| PVESTBR239 | PVEPSE2021E05.seq | (AAC)6 | 361 | CGGGTGTGATGAGTACAGCTAA | TCGTTCTCTTCATTGGAGTTCA | 56 |
| PVESTBR240 | PVEPSE2021H01.seq | (C)6 (A)6 (G)6 (T)6 | 379 | AAGAAAATCCCAGTTCTCCTCC | TTCCATTGATTTGCTCTGTCAC | 56 |
| PVESTBR241 | PVEPSE2021H06.seq | (CT)6 | 149 | GAGGAAAATATGGAGCACTTGG | AATCTACCACACCCCTCTTCAA | 56 |
| PVESTBR242 | PVEPSE2022B10.seq_1 | (T)8 (T)6 (TC)7 | 282 | AGTAAAGCCACTTTCTTCGTGC | CCATTAAACAAATAGAAGCACCC | 56 |
| PVESTBR245 | PVEPSE2022F10.seq | (TC)10 | 106 | TGTAGTTTTCTTTGCCCTTTCC | CTTGGAGTTTCAGAACCAATCC | 56 |
| PVESTBR246 | PVEPSE2023B04.seq | (CCA)7 | 146 | TATCCAAACACAAACCATGTCC | TGGAGATGAGGTTCCTTCTGTT | 56 |
| PVESTBR247 | PVEPSE2025D11.seq | (A)7 (A)7 (A)6 (A)7 | 377 | GGAGTCTTTTCTTTGGATGTGG | AGGTTGAAGCTCCCTCTCTCTT | 56 |
| PVESTBR248 | PVEPSE2026G07.seq | (AGA)6 | 234 | TATCGAAGTCGCTTAGTTGCTG | AAAACGACATTTGTCTTCCGTT | 56 |
| PVESTBR249 | PVEPSE2030D05.seq | (CAT)6 (T)6 | 141 | ACTCTCCCTTTTGATTATCCCC | TTCAGAGTAACAGCACAGGGAA | 56 |
| PVESTBR250 | PVEPSE2031D01.seq | (TTA)6 | 335 | GATTAATTGGTCAAAATGGGTCA | ACAGGAACACCTGATAGGCACT | 56 |
| PVESTBR251 | PVEPSE2032A08.seq | (ACC)6 | 146 | AAACAACCAACACCAACAACAA | GAAGTTGCGGCAGCAGTAGT | 56 |
| PVESTBR252 | PVEPSE2033A05.seq | (CA)6 | 297 | CTTTCTCTGCTCATGCGTGTT | TAGGTCTGAATCGGAACCCTAA | 56 |
| PVESTBR253 | PVEPSE3007B09.seq | (T)7 (T)7 (A)6 (AT)6 (T)6 | 269 | AAGATCCATTACGTGTCCAAGG | AGGAATCGGACTCTAGGAAAGG | 56 |
| PVESTBR254 | PVEPSE3008F01.seq | (TC)7 (C)6 | 184 | ATAAATCAACCCCTGTCACCAC | GGCGTAAGCAGGTGGGTA | 56 |
| PVESTBR255 | PVEPSE3015F02.seq | (AT)6 | 369 | ATCCCTGTCAATAGACCCAATG | CAACCTGTCCGCTTCATATACA | 56 |
| PVESTBR256 | PVEPSE3022E08.seq | (AATG)6 | 203 | TCTTTGATGAAACTGCTGGCTA | ATAATCCATCATGCACCAGCTT | 56 |
| PVESTBR257 | PVEPSE3029M21.seq | (A)7 (A)6 (T)6 (A)7 | 290 | TAAATGCGTGCTGTCACTCTCT | TTCATATTCTAATGTGGTGATTTGTG | 56 |
| PVESTBR258 | PVEPSE3029P24.seq | (AT)9 | 229 | TAGAATTCAAATGTGACACCGC | CAGAACCACCTGAAAGAAAATACA | 56 |
| PVESTBR259 | PVEPSE3030J03.seq | (AGA)6 | 210 | CGTGTGTGTGGTTTTGGTAACT | AAGGTAGAAGAATCCGCATCAA | 56 |
| PVESTBR260 | PVEPSE3030P01.seq | (CGCCAC)6 | 153 | GTTAGATCCCGCCCAATAGTC | GCCGAGGATATCGTAGAGAGAA | 56 |
| PVESTBR261 | RTS_108_D06_046 | (T)6 (T)6 (A)6 (T)6 | 249 | CAATTTCAGACGACAGCTTCAG | GATCAAAACAATCTCAAGAATCCA | 56 |
| PVESTBR262 | RTS_108_H08_064 | (T)7 (T)6 (G)7 (CT)7 (A)6 | 329 | CCCATTCCATCTTGTGATAGGT | ATCCAGCTTGGCTCAGAATAAA | 56 |
| PVESTBR264 | RTS_117_H03_028 | (CAC)6 | 252 | GGTCATGGAACTATTGGGATGT | GACAACAAAAGAAAGGGAATGC | 56 |
| PVESTBR265 | RTS_118_G05_036 | (AAT)7 | 169 | AATCAGTTCAAGACACCAAGAT | TCATTTGGGAAAGGAAACTAT | 56 |
| PVESTBR267 | RTS_120_D02_014 | (T)9 (A)8 (T)7 (T)6 | 261 | AGTGTTGGATCTCGACCTGAAT | AAAAGTTAACAGCAACGGCAAC | 56 |
| PVESTBR268 | RTS_120_D07_058 | (CCA)6 | 289 | AGATGCAGAACAGATGCTTCAA | TGAAAGGGTCTTCTTGTTCCAT | 56 |
| PVESTBR270 | RTS_129_E06_039 | (TAT)7 | 115 | TGGTTAGTTTTGGACACAGTCG | CCAGAGGATACAATAGTGAAGCG | 56 |
| PVESTBR271 | RTS_130_B08_061 | (TA)9 | 165 | ATTTTATCAGCAGCACCACAAA | CCCTGATGCCTACACAGATAAG | 56 |
| PVESTBR272 | RTS_131_D11_090_1 | (AT)8 (A)6 | 165 | GAGAATCATCAGGCACATTTGA | TGAACTTCCCATTGGTTTTACC | 56 |
| PVESTBR273 | RTS_132_D04_030 | (GA)18 (GA)6 | 340 | ATCAAACTAGTGGCAGGTTGGT | TGATGAACCCATTAAAGCATTG | 56 |
| PVESTBR274 | RTS_135_G04_024 | (T)8 (A)7 (T)7 (T)6 | 298 | TGTTCTTTTACGCTCAGCTGTC | ACATTAAAAGCCCTCGCAATAG | 56 |
| PVESTBR275 | RTS_137_G02_008 | (T)6 (T)6 (T)6 (T)6 | 326 | TGCGGGATATACTCAGGAAGAT | ACAACAATGCAGGTATGAAGTTG | 56 |
| PVESTBR277 | RTS_140_D09_074 | (CT)6 (A)7 | 293 | TCAGACTTACAGGCCAGGGTAT | GATCATGGGCAACACTTCAG | 56 |
| PVESTBR278 | RTS_141_A12_085 | (AGA)6 | 373 | AATGAGTCCTCCAACAAGCAAT | GCACCTAACTGTCTGCTTTGTG | 56 |
| PVESTBR279 | RTS_141_B11_089 | (AT)18 | 156 | GCGCAGTACTCATCTTAGCCTT | GGCTTCGCATTTATTGTACCTC | 52 |
| PVESTBR280 | Contig1065.seq-FWD | (A)6 (T)6 (T)7 | 365 | CTCAACAATCAAGGACAGCTTG | GTGCTTGTAATGCCTACCACAA | 56 |
| PVESTBR281 | Contig1123.seq-FWD | (T)7 (T)8 (C)6 | 355 | GGGAACCATCCACCATATAAGA | CCATATCCAACCTACCCAGAAA | 56 |
| PVESTBR282 | Contig1150.seq-FWD | (T)10 (T)7 (A)6 | 163 | AAATACCCAGTTGGTTGGATTG | CAATTTGCTCCTTAAACGTCCT | 56 |
| PVESTBR283 | Contig1168.seq-FWD | (T)8 (T)9 (T)6 | 326 | CCGGATTGATTCTTCTGGTAAC | GAGTGCATACTAGTCCAACCCC | 56 |
| PVESTBR284 | Contig1207.seq-FWD | (A)10 (T)6 (T)8 | 362 | ATCCAGAAAGGAATGACTGGAA | CACATTTATCGCTATCCCAAAA | 56 |
| PVESTBR285 | Contig121.seq-FWD | (T)7 (T)6 (T)8 | 309 | TTTTCACCATTTCCTCGTATCA | GTTTTGGCTTGTCATTTGTTGA | 56 |
| PVESTBR287 | Contig1298.seq-FWD | (T)6 (T)6 (A)8 | 302 | AACCGCTATTGATCTATTCCCA | CAAACATGATTTTCCCTGTTGT | 56 |
| PVESTBR288 | Contig1418.seq-FWD | (T)7 (A)8 (T)8 | 207 | GATCAGCAATGTGTTGAGGTGT | ATTGGTAGGTGGTAGAGAGGCA | 56 |
| PVESTBR289 | Contig145.seq-FWD | (A)6 (T)10 (T)6 | 329 | GACAGGCTTTGTCCTTCAGAGT | CTAATCAACTTCCCCTTGCACT | 56 |
| PVESTBR290 | Contig1518.seq-FWD | (T)6 (A)6 (T)6 | 394 | TTGATGGTTCAAACAACTTTGC | TAACAAATGGACAAGGCAGTTG | 56 |
| PVESTBR292 | Contig1625.seq-FWD | (T)6 (T)9 (T)6 | 311 | CAACCTGTTTCCTCTTATGTTTCA | CACCGTGTGAAAGAACCACTTA | 56 |
| PVESTBR293 | Contig1723.seq-FWD | (T)6 (T)6 (G)6 | 192 | ACACCCCATCCTACAGAAGCTA | AACCACCCTCCTAAAACCTTGT | 56 |
| PVESTBR294 | Contig1737.seq-FWD | (A)7 (C)6 (A)6 | 393 | AGGCTTCGTGGAGTAGAGTCTG | TATGCAATACCCCAATGAATGA | 56 |
| PVESTBR295 | Contig1851.seq-FWD | (A)6 (A)9 (A)7 | 213 | ATCGACACAATATGCCTAGTCG | AAACGTCTACGAAAAGATCGCT | 56 |
| PVESTBR296 | Contig1957.seq-FWD | (T)6 (T)7 (T)6 | 355 | CATGTCGAAGAAACTGAGCAAG | TGCAACTTGTAGCTCCTATCCC | 56 |
| PVESTBR297 | Contig1988.seq-FWD | (A)6 (A)6 (A)7 | 335 | GACCGAAAATGCTATATCAGGC | AAGCCCTCTAAAATTCCCAAAC | 56 |
| PVESTBR298 | Contig2077.seq-FWD | (T)6 (T)8 (T)7 | 298 | GTTTAGGAGTTGAGGCATTTGG | TGAGTTGCATCAATCACAATGA | 56 |
| PVESTBR299 | Contig2090.seq-FWD | (T)7 (T)7 (T)6 | 327 | ATTCATGGAGCTTCTGGACCTA | GTATTCACATCCAGCAAAGCAA | 56 |
| PVESTBR300 | Contig2132.seq-FWD | (T)9 (T)6 (T)10 | 266 | TTTTCAGAATCGAGATCCAGGT | CCATCATAGCTGCCATACAAAA | 56 |
| PVESTBR301 | Contig2178.seq-FWD | (T)7 (T)7 (T)7 | 179 | CCCTTCTCTGTTATTCGATTGG | AATAATGGTAGCATAAAGGGCG | 56 |
| PVESTBR302 | Contig2537.seq-FWD | (T)6 (T)6 (T)7 | 205 | AAAAGTGAGAAAGGATCTTG | GTAAAAGAGAGAATCGAGAAAG | 56 |
| PVESTBR303 | Contig2572.seq-FWD | (T)9 (A)7 (T)6 | 221 | CAATGTTTTCTCCTCCAGAACC | CAATCCTGTGGTGGCTGTATAA | 56 |
| PVESTBR304 | Contig2620.seq-FWD | (T)18 (A)6 (T)7 | 235 | CTTCTCAAGGATAGGCCGTAGA | TTACACTTACATGGTGCCAACC | 56 |
| PVESTBR305 | Contig263.seq-FWD | (A)6 (A)6 (T)7 | 398 | CTCATGCTCAGGATTTTCCTCT | CTCAATTCGTAAAATCGGTTCA | 56 |
| PVESTBR306 | Contig2831.seq-FWD | (T)8 (T)6 (A)6 | 320 | AGGTCACAAAGGAACTAACCCA | TTCCTGAAAAGAAACCATACTGAA | 56 |
| PVESTBR307 | Contig2844.seq-FWD | (T)6 (T)6 (T)7 | 344 | TATGGTTGGTGCTTCAACTCTG | AAAACAAACCCTCCTTTCCCTA | 56 |
| PVESTBR308 | Contig3013.seq-FWD | (T)9 (T)6 (A)7 | 301 | AGCGTGTACTTTAGCAAGAGAGC | TAACAGCAAATGATCTGGGATG | 56 |
| PVESTBR309 | Contig3093.seq-FWD | (C)6 (A)6 (C)6 | 217 | CATTCTGGGCTGTGGCTACT | AGTGACTGGTGGAATAGGCAGT | 56 |
| PVESTBR310 | Contig318.seq-FWD | (T)10 (A)6 (A)6 | 391 | CCACCAAGAGAAAGAAAGAGGA | AAACATTGATGCTGCTACATGC | 56 |
| PVESTBR311 | Contig3200.seq-FWD | (T)6 (A)6 (A)6 | 366 | GCAGACAACCACTGAGACTTTG | AATCCCACCACAAGTCCATAAC | 50 |
| PVESTBR312 | Contig3218.seq-FWD | (A)8 (C)6 (T)6 | 330 | TAAGGGATCTTTTGAGCTCTGG | ATAGCTTCCAATGCAGAATGGT | 56 |
| PVESTBR313 | Contig3230.seq-FWD | (T)7 (T)9 (T)6 | 229 | CCTTAAAGCCGTTCCTATGTTG | GCAGCAGTAAGCAATATTTTCCA | 56 |
| PVESTBR314 | Contig3243.seq-FWD | (A)6 (A)9 (A)10 | 302 | CATTTAAAGGGGATTGGTTGAA | CCCTTAACTACCAGAGCGTTGT | 56 |
| PVESTBR317 | Contig372.seq-FWD | (T)6 (T)7 (A)6 | 272 | CCCTTGTCAGAATTGGAAGAAC | CTTTCATGCTTTCCTCGTTACC | 56 |
| PVESTBR318 | Contig386.seq-FWD | (T)6 (T)6 (T)6 | 237 | AGTACAGCAACAAAACCAGCAA | CACATTGATAGCATCTGGAGGA | 56 |
| PVESTBR319 | Contig456.seq-FWD | (T)9 (A)8 (T)8 | 266 | GAAGATAATTTGACTCCGCCAG | CAAGGAGCACTTCGAGTTATGA | 56 |
| PVESTBR320 | Contig530.seq-FWD | (T)6 (A)8 (A)8 | 332 | AAGTGAAGGGTGATGGTAATGG | TTGGGTTAACTTGTTATAAGCCG | 56 |
| PVESTBR322 | Contig707.seq-FWD | (A)6 (A)6 (A)6 | 284 | AAAACACCAAAATCCACAAACC | TGAGGTAGCTCGACTCAACAAA | 56 |
| PVESTBR323 | Contig73.seq-FWD | (T)6 (A)7 (A)7 | 369 | TGAGACCATCCATTCAATTCAG | TGCTTCATCAAACAGGCTTAAA | 56 |
| PVESTBR325 | Contig80.seq-FWD | (T)6 (A)6 (T)7 | 382 | TCATGGCTCTTGATTCTTGCTA | GGAAAAGCTCTAACTGGCTCAA | 56 |
| PVESTBR326 | Contig863.seq-FWD | (T)6 (T)6 (T)6 | 327 | GGCTTATGCAAACAACTTCCTC | GTGTGAGCATCACCAGAGTTTC | 56 |
| PVESTBR327 | Contig907.seq-FWD | (T)9 (A)6 (T)7 | 393 | AGTTTCTTCTTCCTCAGGGTCC | TTTTCTTTGGGGTCTACTGGTC | 50 |
| PVESTBR329 | Contig980.seq-FWD | (A)7 (A)7 (T)6 | 398 | TGCTCTGCTGTTCAAGAATCAT | AGTTTGGCATCACAACACAGTC | 56 |
| PVESTBR330 | ALV_006_F10_B2-FWD | (A)8 (A)7 (T)7 | 278 | GAAGGACATTTCCCACAAAATC | TTACCCAAGCAATGGAACTTTT | 50 |
| PVESTBR331 | ALV_008_G02_G1-FWD | (T)8 (T)6 (A)6 | 319 | GTTGAGAATTTCCGTTTTCGTC | CTATTTATGCATCACTGGGCAA | 56 |
| PVESTBR333 | ALV_009A_D04_B1-FWD | (T)11 (T)8 (A)7 | 215 | AAAGATGAAGCAGAAGCGGTAG | TGCTCGCAATGGATATTACAAA | 56 |
| PVESTBR334 | ALV_010B_F03_B1-FWD | (T)6 (T)7 (T)7 | 387 | CCACTTAGCTCCACCTCCTCTA | CGTGGAAGCCAAAATATTGATAC | 50 |
| PVESTBR335 | ALV_010C_C05_B1-FWD | (T)6 (T)7 (T)7 | 268 | AGGCTGTGACCACTGATTTTCT | ATCTCAAAACAACGTGAACCCT | 56 |
| PVESTBR336 | ALV_011A_G01_B1-FWD | (T)11 (A)6 (T)8 | 332 | TGTTTTGGTACCCGAATCACTA | CAATTCATCCAAAATGAACAAAC | 50 |
| PVESTBR337 | ALV_011B_F05_B1-FWD | (A)6 (A)6 (T)8 | 379 | CAAATCCTTTTGAACCAACCAT | GTCTTCATAATGCAACCACCCT | 50 |
| PVESTBR338 | ALV_011D_E02_B1-FWD | (A)6 (A)7 (A)6 | 245 | GCTAACCAGATACCAAAGCACC | AGACAAACAATGATTCCCAACC | 56 |
| PVESTBR340 | ALV_012C_F02_B1-FWD | (A)6 (T)7 (T)6 | 371 | ATTACAGGCAGCATCAACTCCT | CTTCAACATGAAAGCCTGAACA | 56 |
| PVESTBR341 | ALV_014C_E10_B1-FWD | (T)6 (T)6 (A)6 | 283 | TGTCAGAACTTTTCTTTGTCGG | GGTTATGAGTGTGTCACTTTTGGA | 56 |
| PVESTBR342 | ALV_016B_B10_B1-FWD | (T)8 (T)6 (T)7 | 328 | GTCTCAAAAGCCCAGAAGAAGA | CCTGGCAAACCCTATATTTTCA | 50 |
| PVESTBR343 | ALV_016B_E03_B1-FWD | (A)6 (T)6 (T)6 | 303 | CCAAGCACACATGTTAAGGAAA | CATGATTGGGCCATTAGAAAAT | 50 |
| PVESTBR344 | LVS_005_D05_b2-FWD | (T)6 (T)6 (T)7 | 250 | CTTATTGAATGAATCTGTGCCG | AAAGTGTAACGCCATCCAAACT | 56 |
| PVESTBR345 | LVS_005_E03_b2-FWD | (T)6 (T)7 (T)9 | 181 | ATTCTCTCCCATTCTCCTCCTT | TGTGACAAATCCTGTTTTGCTT | 56 |
| PVESTBR347 | LVS_040_C11_b1-FWD | (T)10 (T)10 (T)6 | 201 | CAAGGCTAATAACAAGATTT | CAACTTTGTGATGAATTAAG | 48 |
| PVESTBR348 | LVS_048_D08_b1-FWD | (A)6 (A)6 (G)6 | 270 | TAAGCCTTGTTTCTTGAGGAGG | CTACTTCCTCAGGGTTTGATGG | 56 |
| PVESTBR349 | NOD_220_D11_091-FWD | (C)6 (A)6 (A)6 | 208 | CACCGACCCCTATGTCTTCTC | GGATGAGTTTATGAGGAGTGGC | 56 |
| PVESTBR350 | NOD_239_C12_087-FWD | (A)9 (T)6 (T)6 | 172 | GAAACTGGAGCCTCAATTCATC | ACAAAAGAATCGCGAATACGAT | 48 |
| PVESTBR351 | NOD_242_B08_061-FWD | (T)8 (T)9 (T)7 | 368 | CCCTGATTTTGAATACTAGCCG | TCATTCCGTCCCCAAGTATAAC | 56 |
| PVESTBR352 | NOD_242_C12_086-FWD | (A)7 (A)6 (T)7 | 328 | AACTCTTTGTCTTCTCGGTTGC | AGCCTGACATTCATCGCATAG | 50 |
| PVESTBR355 | NOD_243_H08_064-FWD | (T)6 (T)7 (T)7 | 371 | TGCATATACCGGAAACACTCTG | CAATTATCGTTCCCATCACCTT | 56 |
| PVESTBR357 | POD_019_H02_016-FWD | (T)6 (A)7 (T)6 | 395 | TCTGAATCTGCCATTTCTGCTA | TCCCAATAATAAACGCATTCAA | 50 |
| PVESTBR358 | POD_022_C04_022-FWD | (T)7 (A)6 (T)6 | 333 | TTGAGGTCACAAGCTAAAGCAG | GCAAAAGAAAGAAGCAGGAAAA | 56 |
| PVESTBR359 | POD_029_G06_040-FWD | (T)6 (T)6 (A)10 | 146 | AGCATTTCATTGGTGCAAGTTA | CTGGAGAACAACAAAATTGACG | 56 |
| PVESTBR360 | POD_031_B09_073-FWD | (T)6 (A)6 (T)7 | 400 | GTGTAAAACCATCTCCTCTGCC | CTGCACAAAACAACAAATCCAT | 56 |
| PVESTBR361 | POD_034_A09_065-FWD | (T)9 (T)6 (T)8 | 267 | GTACTCCTTCCGTCACTCCTTC | TCCAAAGAATGAACTCTCACACA | 56 |
| PVESTBR362 | POD_035_A07_049-FWD | (T)6 (T)7 (T)6 | 278 | CGGAGAAGGTGAAAAGAAGAGA | CTTTCAGCACGGGTAAACTTCT | 56 |
| PVESTBR363 | POD_040_F06_047-FWD | (A)7 (A)6 (T)6 | 230 | GGACCTGCAAAAGAAGAGAAGA | CAGTTGCTTCTGAATATGGGAA | 56 |
| PVESTBR364 | PVEPSE2011B03.seq-FWD | (T)6 (T)10 (A)7 | 382 | GCTGGTATGAGTTTTCTATTTGACG | ATCTCGAACCCAACTTCCACTA | 56 |
| PVESTBR365 | PVEPSE2012G11.seq-FWD | (T)7 (T)6 (T)6 | 323 | GAATTGGGTTTCAAGTTTCTGC | CTTTCCACTTACAACCTTTCCG | 56 |
| PVESTBR367 | PVEPSE2014G10.seq-FWD | (A)6 (T)6 (A)6 | 196 | AGCAAGAAACGCATATACAGCA | CGATCCACACGTTTCAATCTTA | 56 |
| PVESTBR368 | PVEPSE2015D05.seq-FWD | (T)9 (T)11 (T)8 | 250 | CCCGTCCCTGTATTAGTGACAT | ATCCTAAACCCATGTTAGGCAG | 56 |
| PVESTBR369 | PVEPSE2129D01.seq-FWD | (A)7 (G)9 (A)7 | 213 | ACGTTTCCAACCGTACAGAAGT | AGCAGGGATGCTGTTTAACCTA | 56 |
| PVESTBR370 | PVEPSE3028O19.seq-FWD | (T)6 (A)6 (A)6 | 226 | GAGGAACAAGAGCATCTCCAAT | AATTTGCACTGAATTTACGCCT | 56 |
| PVESTBR371 | RTS_109_B07_057-FWD | (A)6 (C)6 (T)6 | 363 | TGACGAGAACGTAAATCAGCTC | TTTCAATCAGAAAACCCTGCTT | 48 |
| PVESTBR372 | RTS_120_C11_082-FWD | (T)6 (T)7 (T)7 | 348 | CAGAGAGGGACAGAGGTTGAGT | AATTGCAGTTTCTTTGGCCTTA | 56 |
| PVESTBR373 | RTS_128_F02_015-FWD | (T)7 (T)9 (T)7 | 234 | CACCACTTGTTGAGCATGATTT | GCAGCTCGGTAGCATTTATTG | 56 |
| PVESTBR374 | RTS_128_F03_027-FWD | (A)6 (A)6 (T)7 | 142 | ACAAGGTCAACCACCTCAGTG | GATAACGAGGACCAAGCAGAAG | 56 |
| PVESTBR375 | RTS_138_H04_032-FWD | (T)6 (A)8 (A)7 | 376 | TTATCCCATCAAAGCTCATCCT | TTTAGATGCTGATTTCCCCAAT | 56 |
| PVESTBR377 | RTS_144_G06_040-FWD | (A)6 (T)10 (T)6 | 389 | TTCCTCAGTCTAAACCTCCCAA | CCCCTCTTAATGGGAAGAGAGT | 56 |
