# Supplementary material for: The characterization of a new set of EST-derived simple sequence repeat (SSR) markers as a resource for the genetic analysis of Phaseolus vulgaris
Source: BMC Genet. 2011 May 9;12:41. doi: 10.1186/1471-2156-12-41 (PMC3102039; doi:10.1186/1471-2156-12-41)
Supplement: Additional File 2 — Table S2. Cross-amplification of 167 SSR markers on 11 Legume species. The expected allele size (EAS) for each marker is indicated. [file 1471-2156-12-41-S2.DOC]

**Table S2. Cross-amplification of 167 SSR markers on 11 Legume species.** The expected allele size (EAS) for each marker is indicated.

| **Locus** | **EAS** | Phaseolus vulgaris | ***Medicago sativa*** | ***P. lunatus*** | ***P. coccineus*** | ***P. acutifolius*** | ***Vigna mungo*** | ***V. angularis*** | ***V. unguiculata*** | ***Glycine max*** | ***Arachis hypogaea*** | ***Dipteryx alata*** | **Origem** |
| --- | --- | --- | --- | --- | --- | --- | --- | --- | --- | --- | --- | --- | --- |
| **AG1** | 132 | 140/155 (A**^1^**) | -/- | 175/165 (A) | 155/158 (A) | 158/155 (A) | 130/130 (A) | 148/148 (A) | 158/155 (A) | -/- | -/- | -/- | Genomic |
| **AJ416389** | 192 | 200/198 (A) | -/- | -/- | -/- | -/- | -/- | -/- | -/- | -/- | -/- | -/- | Genomic |
| **AJ416391** | 186 | 195/195 (A) | -/- | -/- | -/- | -/- | -/- | -/- | -/- | -/- | -/- | -/- | Genomic |
| **AJ416395** | 242 | 250/250 (A) | -/- | -/- | -/- | -/- | -/- | -/- | -/- | -/- | -/- | -/- | Genomic |
| **BM3** | 193 | 193/193 (A) | -/- | 193/193 (A) | -/- | 193/193 (B) | -/- | -/- | -/- | -/- | -/- | -/- | Genomic |
| **BM6** | 153 | 155/155 (A) | -/- | -/- | 155/155 (A) | 165/160 (A) | -/- | -/- | -/- | -/- | -/- | -/- | Genomic |
| **BM16** | 149 | 170/170 (A) | 160/160 (D) | 160/160 (D) | 180/160 (D) | 160/160 (D) | -/- | -/- | -/- | -/- | -/- | -/- | Genomic |
| **BM20** | 146 | 180/180 (B) | -/- | -/- | -/- | -/- | -/- | -/- | -/- | -/- | -/- | -/- | Genomic |
| **BM68** | 170 | 180/180 (A) | -/- | 175/175 (B) | -/- | 180/180 (B) | -/- | -/- | -/- | -/- | -/- | -/- | Genomic |
| **BM98** | 247 | 240/245 (A) | 245/245 (A) | 240/245 (A) | 245/215 (A) | 240/245 (A) | 230/245 (A) | 230/235/245 (A) | -/- | 215/245 (A) | 245/245 (A) | 245/245 (A) | Genomic |
| **BM114** | 234 | 250/280 (A) | -/- | -/- | -/- | -/- | -/- | -/- | -/- | -/- | -/- | -/- | Genomic |
| **BM137** | 155 | 115/115 (A) | -/- | -/- | 115/115 (B) | -/- | -/- | -/- | -/- | -/- | -/- | -/- | Genomic |
| **BM138** | 203 | 210/210 (A) | -/- | -/- | -/- | -/- | -/- | -/- | -/- | -/- | -/- | -/- | Genomic |
| **BM140** | 190 | 160/160 (A) | 168/168 (A) | 170/170 (A) | -/- | 150/150 (A) | -/- | -/- | -/- | -/- | -/- | -/- | Genomic |
| **BM142** | 157 | 165/162 (A) | -/- | 157/157 (A) | 147/147 (A) | 160/160 (A) | -/- | -/- | 143/143 (A) | -/- | -/- | -/- | Genomic |
| **BM143** | 143 | 205/285 (A) | -/- | 155/155 (A) | -/- | 183/183 (A) | -/- | -/- | -/- | -/- | -/- | -/- | Genomic |
| **BM146** | 281 | 285/270 (B) | -/- | 280/280 (B) | 278/278 (B) | 285/290 (B) | -/- | 310/310 (B) | 285/285 (B) | -/- | -/- | -/- | Genomic |
| **BM148** | 295 | 310/310 (A) | -/- | 330/330 (A) | 310/310 (B) | 310/310 (B) | -/- | -/- | 310/310 (B) | -/- | -/- | -/- | Genomic |
| **BM149** | 273 | 245/248 (A) | -/- | 253/253 (A) | -/- | 240/240 (A) | 268/268 (A) | 258/258 (A) | -/- | -/- | -/- | -/- | Genomic |
| **BM151** | 153 | 152/148 (A) | -/- | 138/138 (A) | 149/149 (A) | 138/138 (A) | -/- | -/- | -/- | -/- | -/- | -/- | Genomic |
| **BM153** | 226 | 229/229 (A) | -/- | -/- | -/- | -/- | -/- | -/- | -/- | -/- | -/- | -/- | Genomic |
| **BM154** | 218 | 230/210 (A) | -/- | 205/205 (A) | -/- | 218/218 (A) | -/- | -/- | -/- | -/- | -/- | -/- | Genomic |
| **BM155** | 114 | 122/122 (A) | -/- | -/- | -/- | 115/115 (B) | -/- | -/- | -/- | -/- | -/- | -/- | Genomic |
| **BM157** | 113 | 100/118 (B) | 112/112 (B) | 112/112 (B) | 112/112 (B) | -/- | -/- | -/- | 114/115 (B) | 115/115 (B) | -/- | -/- | Genomic |
| **BM158** | 130 | 102/102 (B) | -/- | -/- | 102/102 (B) | -/- | 102/102 (B) | 102/102 (B) | -/- | -/- | -/- | -/- | Genomic |
| **BM159** | 198 | 220/220 (A) | -/- | -/- | -/- | 205/208 (A) | -/- | -/- | -/- | -/- | -/- | -/- | Genomic |
| **BM160** | 211 | 218/225 (A) | -/- | 190/190 (A) | -/- | 190/190 (A) | -/- | -/- | -/- | -/- | -/- | -/- | Genomic |
| **BM161** | 185 | 195/195 (A) | -/- | 190/190 (A) | 198/198 (A) | -/- | 138/138 (A) | 130/130 (A) | 198/198 (A) | -/- | -/- | -/- | Genomic |
| **BM164** | 182 | 210/200 (B) | -/- | 158/158 (B) | 160/160 (B) | 153/153 (B) | -/- | -/- | 155/155 (B) | -/- | -/- | 180/180 (B) | Genomic |
| **BM165** | 177 | 178/178 (A) | -/- | -/- | -/- | -/- | -/- | -/- | -/- | -/- | -/- | -/- | Genomic |
| **BM166** | 151 | 165/165 (A) | -/- | 160/160 (A) | -/- | 160/160 (A) | 138/138 (A) | 138/138 (A) | -/- | -/- | -/- | -/- | Genomic |
| **BM167** | 165 | 108/108 (A) | -/- | -/- | 109/109 (A) | 102/102 (A) | -/- | -/- | 109/109 (A) | -/- | -/- | -/- | Genomic |
| **BM175** | 170 | 180/175 (A) | -/- | -/- | -/- | 150/150 (A) | 115/115 (A) | -/- | -/- | -/- | -/- | -/- | Genomic |
| **BM181** | 192 | 180/180 (A) | -/- | 180/170 (A) | -/- | 175/173 (A) | -/- | -/- | -/- | -/- | -/- | -/- | Genomic |
| **BM183** | 149 | 152/152 (A) | -/- | 145/145 (A) | 137/137 (A) | 155/155 (A) | 132/132 (A) | 132/132 (A) | 137/137 (A) | 162/162 (A) | -/- | -/- | Genomic |
| **BM184** | 160 | 165/165 (A) | -/- | -/- | -/- | -/- | -/- | -/- | -/- | -/- | -/- | -/- | Genomic |
| **BM185** | 105 | 108/108 (A) | 105/105 (A) | 100/100 (A) | 105/105 (A) | 100/100 (A) | 107/107 (A) | -/- | 105/105 (A) | 107/107 (A) | -/- | -/- | Genomic |
| **BM187** | 191 | 170/158 (A) | 162/162 (C) | 162/162 (C) | 132/132 (A) | 98/98 (A) | -/- | -/- | -/- | -/- | -/- | -/- | Genomic |
| **BM189** | 114 | 127/125 (B) | -/- | 102/98 (B) | -/- | 115/114 (B) | -/- | -/- | -/- | -/- | -/- | -/- | Genomic |
| **BM197** | 201 | 205/205 (A) | -/- | 205/203 (A) | 190/190 (A) | 208/208 (A) | 195/195 (A) | 193/193 (A) | 193/193 (A) | 215/215 (A) | -/- | -/- | Genomic |
| **BM200** | 221 | 238/238 (B) | -/- | 225/225 (B) | -/- | 215/215 (B) | -/- | -/- | -/- | -/- | -/- | -/- | Genomic |
| **BM201** | 102 | 189/193 (A) | -/- | 205/205 (A) | -/- | 193/193 (A) | -/- | -/- | -/- | -/- | -/- | -/- | Genomic |
| **BM202** | 156 | 160/160 (A) | -/- | 142/145 (A) | 130/130 (A) | 140/140 (A) | -/- | -/- | -/- | -/- | -/- | -/- | Genomic |
| **BM205** | 137 | 138/135 (A) | -/- | -/- | -/- | -/- | -/- | -/- | -/- | -/- | -/- | -/- | Genomic |
| **BM210** | 166 | 172/170 (A) | -/- | 151/151 (A) | -/- | 148/148 (A) | -/- | -/- | -/- | -/- | -/- | -/- | Genomic |
| **BM211** | 186 | 205/208 (A) | -/- | 218/218 (B) | -/- | 205/205 (B) | -/- | -/- | -/- | -/- | -/- | -/- | Genomic |
| **BM212** | 214 | 212/232 (A) | -/- | -/- | -/- | -/- | -/- | -/- | -/- | -/- | -/- | -/- | Genomic |
| **BMc78** |  | 158/158 (A) | -/- | 152/152 (A) | -/- | 148/148 (A) | -/- | -/- | -/- | -/- | -/- | -/- | EST |
| **BMd64-1** |  | 128/128 (A) | 130/130 (A) | 130/132 (A) | 132/128 (A) | 128/128 (A) | -/- | -/- | -/- | -/- | -/- | -/- | EST |
| **GATs11B** | 160 | 167/165 (A) | -/- | -/- | -/- | 170/168 (A) | -/- | -/- | -/- | -/- | -/- | -/- | Genomic |
| **GATs54** | 114 | 127/130 (B) | -/- | 122/122 (B) | -/- | -/- | -/- | -/- | -/- | -/- | -/- | -/- | Genomic |
| **PV5** | 195 | 180/180 (A) | -/- | -/- | -/- | -/- | -/- | -/- | -/- | -/- | -/- | -/- | Genomic |
| **PV11** | 142 | 189/189 (A) | -/- | 175/175 (A) | -/- | 187/190 (A) | -/- | -/- | -/- | -/- | -/- | -/- | Genomic |
| **PV12** | 193 | 190/190 (A) | -/- | 172/172 (A) | -/- | 187/187 (A) | -/- | -/- | -/- | -/- | -/- | -/- | Genomic |
| **PV13** | 196 | 198/198 (A) | -/- | -/- | -/- | -/- | -/- | -/- | -/- | -/- | -/- | -/- | Genomic |
| **PV25** | 158 | 175/150 (A) | -/- | 138/128 (A) | -/- | 135/138 (A) | -/- | -/- | -/- | -/- | -/- | -/- | Genomic |
| **PV35** | 214 | 215/245 (A) | -/- | 210/210 (A) | -/- | 215/215 (A) | -/- | -/- | -/- | -/- | -/- | -/- | Genomic |
| **PV38** |  | 220/220 (B) | -/- | -/- | -/- | -/- | -/- | -/- | -/- | -/- | -/- | -/- | Genomic |
| **PV51** |  | 156/156 (A) | -/- | 158/156 (A) | 157/157 (A) | 156/156 (A) | 158/158 (A) | 163/163 (A) | 156/156 (A) | -/- | -/- | -/- | Genomic |
| **PV53** | 165 | 172/175 (A) | -/- | 175/175 (B) | 175/175 (B) | 180/180 (B) | 190/190 (B) | -/- | -/- | -/- | -/- | -/- | Genomic |
| **PV55** |  | 242/243 (A) | -/- | 245/245 (C) | -/- | 242/242 (C) | -/- | -/- | -/- | -/- | -/- | -/- | Genomic |
| **PV60** | 171 | 162/162 (B) | -/- | 180/180 (B) | 162/162 (B) | 150/150 (B) | -/- | -/- | -/- | -/- | -/- | -/- | Genomic |
| **PV67** | 152 | 145/145 (B) | -/- | -/- | -/- | -/- | -/- | -/- | -/- | -/- | -/- | -/- | Genomic |
| **PV77** |  | 235/248 (A) | -/- | 268/268 (A) | 250/250 (B) | 252/252 (B) | -/- | -/- | -/- | -/- | -/- | -/- | Genomic |
| **PV80** |  | 208/208 (A) | 280/280 (B) | 310/310 (B) | 208/208 (B) | 260/260 (B) | -/- | -/- | -/- | -/- | -/- | -/- | Genomic |
| **PV87** | 163 | 188/205 (B) | -/- | 153/160 (B) | -/- | 160/153 (B) | -/- | -/- | -/- | -/- | -/- | -/- | Genomic |
| **PV96** |  | 162/162 (A) | -/- | -/- | 162/162 (A) | -/- | -/- | -/- | -/- | -/- | -/- | -/- | Genomic |
| **PV97** |  | 134/134 (B) | -/- | 130/130 (B) | 125/130 (B) | -/- | 132/132 (B) | 132/132 (B) | 125/125 (B) | 140/140 (B) | -/- | -/- | Genomic |
| **PV101** | 150 | 185/185 (B) | -/- | -/- | -/- | -/- | -/- | -/- | -/- | 190/190 | -/- | -/- | Genomic |
| **PV102** | 181 | 190/190 (B) | -/- | -/- | -/- | -/- | -/- | -/- | -/- | -/- | -/- | -/- | Genomic |
| **PV105** |  | 210/210 (B) | -/- | -/- | -/- | -/- | -/- | -/- | -/- | -/- | -/- | -/- | Genomic |
| **PV112** | 183 | 187/190 (A) | -/- | 173/179 (A) | 187/187 (A) | 180/180 (A) | -/- | -/- | -/- | -/- | -/- | -/- | Genomic |
| **PV113** | 100 | 100/100 (B) | -/- | 97/95 (B) | -/- | 93/93 (B) | -/- | -/- | -/- | -/- | -/- | -/- | Genomic |
| **PV118** |  | 248/248 (A) | -/- | 230/230 (A) | -/- | 238/238 (B) | -/- | -/- | -/- | -/- | -/- | -/- | Genomic |
| **PV131** | 198 | 120/120 | -/- | -/- | -/- | 115/115 | -/- | -/- | -/- | -/- | -/- | -/- | Genomic |
| **PV140** |  | 220/220 (A) | -/- | 220/220 (A) | 220/220 (A) | 225/225 (A) | 220/220 (B) | 220/220 (B) | 220/220 (B) | 220/220 (B) | -/- | 220/220 (B) | Genomic |
| **PV148** |  | 222/220 (A) | -/- | -/- | 220/220 (B) | -/- | 280/280 (B) | -/- | -/- | -/- | -/- | -/- | Genomic |
| **PV162** |  | 225/225 (A) | -/- | -/- | -/- | -/- | -/- | -/- | -/- | -/- | -/- | -/- | Genomic |
| **PV163** | 217 | 255/310 (A) | -/- | 215/215 (A) | -/- | 240/240 (A) | -/- | -/- | -/- | -/- | -/- | -/- | Genomic |
| **PV168** | 190 | 195/195 (B) | -/- | 198/198 (B) | -/- | -/- | -/- | -/- | -/- | -/- | -/- | -/- | Genomic |
| **PV169** | 205 | 230/230 (B) | -/- | -/- | -/- | -/- | -/- | -/- | -/- | -/- | -/- | -/- | Genomic |
| **PV174** |  | 200/202 (A) | -/- | 130/130 (B) | -/- | 196/196 (B) | -/- | -/- | -/- | -/- | -/- | -/- | Genomic |
| **PV180** |  | 150/152 (B) | -/- | 147/147 (B) | -/- | 152/152 (B) | -/- | -/- | -/- | -/- | -/- | -/- | Genomic |
| **PV193** |  | 165/165 (B) | -/- | 290/290 (B) | 300/300 (B) | 288/288 (B) | -/- | -/- | -/- | -/- | -/- | -/- | Genomic |
| **PV194** |  | 210/210 (B) | 250/250 (B) | 212/212 (B) | 220/220 (B) | 212/212 (B) | -/- | -/- | -/- | 245/245 (B) | -/- | 280/280 (B) | Genomic |
| **PV198** | 222 | 242/242 (A) | -/- | -/- | -/- | -/- | -/- | -/- | -/- | -/- | -/- | -/- | Genomic |
| **PV200** |  | 230/230 (B) | -/- | -/- | -/- | -/- | -/- | -/- | -/- | -/- | -/- | -/- | Genomic |
| **PV202** |  | 233/248 (A) | -/- | 265/258 (A) | 250/250 (A) | 252/250 (A) | -/- | -/- | -/- | 230/210 230/225 (B) | -/- | -/- | Genomic |
| **PV204** |  | 187/187 (B) | -/- | -/- | 187/187 (B) | -/- | -/- | -/- | -/- | -/- | -/- | -/- | Genomic |
| **PV207** |  | 195/195 (A) | -/- | -/- | 195/195 (B) | -/- | -/- | -/- | -/- | -/- | -/- | -/- | Genomic |
| **PV215** | 220 | 250/250 (A) | -/- | -/- | -/- | -/- | -/- | -/- | -/- | -/- | -/- | -/- | Genomic |
| **PV221** |  | 180/180 (B) | -/- | -/- | -/- | -/- | -/- | -/- | -/- | -/- | -/- | -/- | Genomic |
| **PV231** |  | 180/180 (A) | -/- | 190/190 (A) | -/- | 175/175 (A) | -/- | -/- | -/- | -/- | -/- | -/- | Genomic |
| **PV237** |  | 165/165 (A) | -/- | -/- | 165/165 (B) | -/- | -/- | -/- | -/- | -/- | -/- | -/- | Genomic |
| **PV243** |  | 230/230 (A) | -/- | -/- | -/- | -/- | -/- | -/- | -/- | -/- | -/- | -/- | Genomic |
| **PV251** | 204 | 205/208 (A) | 248/248 (B) | 198/199 (A) | 208/208 (A) | 198/198 (A) | -/- | -/- | 220/220 (B) | -/- | -/- | -/- | Genomic |
| **PV254** |  | 225/225 (B) | -/- | -/- | -/- | -/- | -/- | -/- | -/- | -/- | -/- | -/- | Genomic |
| **PV258** |  | 270/270 (A) | -/- | -/- | -/- | 310/310 (B) | -/- | -/- | -/- | -/- | -/- | -/- | Genomic |
| **PV259** | 127 | 140/140 (B) | -/- | -/- | -/- | -/- | -/- | -/- | -/- | -/- | -/- | -/- | Genomic |
| **PV265** |  | 145/145 (A) | -/- | -/- | -/- | -/- | -/- | -/- | -/- | -/- | -/- | -/- | Genomic |
| **PV268** |  | 95/95 (B) | -/- | -/- | -/- | -/- | -/- | -/- | -/- | -/- | -/- | -/- | Genomic |
| **PV270** |  | 123/123 (A) | -/- | 121/121 (A) | -/- | 125/125 (A) | -/- | -/- | -/- | -/- | -/- | -/- | Genomic |
| **PV272** |  | 112/110 (B) | -/- | -/- | 118/118 (B) | -/- | -/- | -/- | -/- | -/- | -/- | -/- | Genomic |
| **PV273** |  | 155/155 (A) | -/- | -/- | -/- | -/- | -/- | -/- | -/- | -/- | -/- | -/- | Genomic |
| **PVEST1** | 299 | 310/310 (A) | -/- | 270/270 (A) | -/- | 290/320 (A) | -/- | -/- | -/- | -/- | -/- | -/- | EST |
| **PVEST6** | 151 | 150/120 (B) | -/- | 102/95 (B) | 90/90 (B) | 85/85 (B) | -/- | -/- | 90/90 (B) | -/- | -/- | -/- | EST |
| **PVEST7** | 230 | 220/230 (B) | -/- | -/- | -/- | -/- | -/- | -/- | -/- | -/- | -/- | -/- | EST |
| **PVEST8** | 188 | 180/180 (A) | -/- | 175/170 (A) | 178/180 (A) | 163/165 (A) | 165/165 (A) | 160/160 (A) | 163/163 (A) | 163/163 (A) | -/- | -/- | EST |
| **PVEST10** | 269 | 250/248 (A) | -/- | 250/250 (A) | 230/230 (A) | 248/248 (A) | 230/230 (A) | -/- | 230/230 (A) | 230/248 (A) | -/- | -/- | EST |
| **PVEST17** | 198 | 198/198 (A) | 320/320 (A) | 220/220 (A) | 235/235 (A) | 192/192 (A) | 185/185 (B) | -/- | 185/185 (A) | -/- | -/- | 195/195 (B) | EST |
| **PVEST23** | 361 | 360/360 (A) | -/- | 360/360 (A) | -/- | 360/360 (A) | -/- | -/- | -/- | 360/360 (B) | -/- | -/- | EST |
| **PVEST26** | 343 | 360/360 (A) | -/- | 360/360 (A) | 380/380 (A) | 370/380 (A) | 380/380 (A) | -/- | 380/380 (B) | -/- | -/- | -/- | EST |
| **PVEST29** | 143 | 155/160 (B) | -/- | 165/172 (B) | -/- | 155/155 (B) | -/- | -/- | -/- | -/- | -/- | -/- | EST |
| **PVEST30** | 188 | 198/198 (A) | -/- | 230/220 (C) | 195/195 (A) | 250/240 (A) | 198/198 (A) | -/- | 190/190 (B) | 188/188 (B) | 190/190 (B) | 198/198 (B) | EST |
| **PVEST34** | 196 | 195/193 (A) | -/- | 200/200 (A) | 210/210 (A) | 193/193 (A) | -/- | 210/210 (A) | 210/210 (A) | -/- | -/- | -/- | EST |
| **PVEST42** | 177 | 170/160 (A) | -/- | -/- | -/- | -/- | -/- | -/- | -/- | -/- | -/- | -/- | EST |
| **PVEST49** | 302 | 290/290 (A) | -/- | 290/290 (A) | -/- | 295/300 (A) | 320/320 (A) | -/- | -/- | -/- | -/- | -/- | EST |
| **PVEST55** | 308 | 300/295 (A) | -/- | 250/250 (A) | 300/300 (A) | 280/280 (A) | 310/310 (A) | 280/280 (B) | 300/300 (B) | -/- | -/- | -/- | EST |
| **PVEST61** | 137 | 130/130 (A) | -/- | 135/135 (A) | 135/135 (A) | 130/130 (A) | 127/127 (A) | -/- | -/- | -/- | -/- | -/- | EST |
| **PVEST71** | 112 | 150/150 (B) | -/- | 140/140 (B) | 145/145 (B) | 135/135 (B) | -/- | -/- | 140/140 (B) | -/- | -/- | -/- | EST |
| **PVEST72** | 219 | 165/170 (B) | -/- | 150/150 (B) | 165/200 (B) | -/- | -/- | -/- | 160/160 (B) | -/- | -/- | -/- | EST |
| **PVEST73** | 150 | 162/162 (A) | -/- | 162/164 (A) | 105/105 (A) | 162/162 (A) | 153/153 (A) | -/- | 105/105 (B) | 105/105 (B) | -/- | 150/150 (B) | EST |
| **PVEST86** | 147 | 155/155 (A) | -/- | 150/150 (A) | 150/150 (A) | 150/150 (A) | 160/160 (A) | 150/150 (A) | 150/150 (A) | 150/150 (A) | -/- | -/- | EST |
| **PVEST98** | 134 | 125/125 (A) | -/- | 135/135 (A) | -/- | 130/115 (A) | 120/120 (A) | -/- | 120/120 (A) | -/- | -/- | -/- | EST |
| **PVEST99** | 168 | 160/160 (A) | -/- | 155/155 (A) | 160/160 (A) | 158/158 (A) | 158/158 (A) | 158/158 (A) | 160/160 (A) | 158/158 (A) | -/- | -/- | EST |
| **PVEST101** | 296 | 340/330 (A) | -/- | 310/310 (A) | 300/300 (A) | 310/310 (A) | -/- | 340/340 (A) | 310/310 (A) | -/- | -/- | -/- | EST |
| **PVEST106** | 338 | 450/450 (A) | 450/430 (D) | 430/430 (A) | 450/430 (A) | 450/450 (A) | -/- | -/- | -/- | -/- | -/- | -/- | EST |
| **PVEST107** | 391 | 470/470 (A) | 480/480 (D) | 470/470 (C) | 460/460 (A) | 460/460 (A) | 460/460 (B) | 310/310 (A) | 310/310 (A) | 350/350 (A) | -/- | -/- | EST |
| **PVEST112** | 318 | 500/500 (A) | -/- | 460/460 (A) | 480/480 (A) | 500/500 (A) | -/- | -/- | 500/500 (A) | -/- | -/- | -/- | EST |
| **PVEST114** | 143 | 145/145 (A) | -/- | 145/145 (A) | -/- | 150/150 (A) | 135/135 (A) | -/- | -/- | -/- | -/- | -/- | EST |
| **PVEST120** | 229 | 230/230 (A) | -/- | 235/235 (A) | 233/233 (A) | 230/230 (A) | -/- | -/- | -/- | -/- | -/- | -/- | EST |
| **PVEST121** | 340 | 350/350 (A) | -/- | 352/352 (A) | 352/352 (B) | 352/352 (A) | -/- | 340/340 (B) | -/- | 340/340 (B) | -/- | -/- | EST |
| **PVEST127** | 292 | 300/300 (A) | 300/300 (A) | 320/320 (A) | 305/305 (A) | 305/305 (A) | 290/290 (A) | 300/300 (A) | 300/300 (A) | 295/295 (A) | -/- | 290/290 (A) | EST |
| **PVEST137** | 185 | 210/185 (A) | -/- | 123/125 (A) | 148/148 (A) | 175/175 (A) | 150/150 (A) | 150/150 (A) | 148/148 (A) | 148/148 (A) | -/- | -/- | EST |
| **PVEST138** | 341 | 340/340 (A) | -/- | 330/330 (A) | 340/340 (C) | 340/340 (A) | 350/350 (A) | -/- | 360/360 (A) | 360/360 (B) | -/- | -/- | EST |
| **PVEST144** | 383 | 340/340 (A) | -/- | 340/340 (A) | -/- | 340/340 (A) | -/- | -/- | -/- | -/- | -/- | -/- | EST |
| **PVEST147** | 259 | 245/250 (A) | -/- | 260/250 (A) | 250/250 (A) | 270/270 (A) | -/- | -/- | -/- | -/- | -/- | -/- | EST |
| **PVEST161** | 252 | 290/290 (A) | -/- | 280/280 (A) | 285/285 (A) | 280/280 (A) | -/- | -/- | -/- | 290/292 (A) | -/- | -/- | EST |
| **PVEST164** | 315 | 330/330 (A) | -/- | 330/330 (A) | 350/350 (A) | 330/330 (A) | 330/330 (A) | 330/330 (A) | 340/340 (B) | -/- | -/- | -/- | EST |
| **PVEST166** | 136 | 138/138 (B) | -/- | 122/122 (B) | 118/118 (B) | 150/150 (B) | -/- | -/- | -/- | 118/118 (B) | -/- | -/- | EST |
| **PVEST168** | 153 | 153/153 (B) | -/- | 147/147 (B) | 153/153 (B) | 148/150 (B) | 178/178 (B) | 172/172 (B) | 153/153 (B) | -/- | -/- | -/- | EST |
| **PVEST186** | 208 | 212/212 (A) | -/- | 212/212 (A) | -/- | 212/212 (A) | -/- | -/- | -/- | 212/212 (A) | -/- | -/- | EST |
| **PVEST195** | 194 | 340/340 (A) | 340/340 (A) | 350/340 340 (A) | 360/340 360/340 (A) | 340/340 (A) | 350/340 350/340 (A) | 350/340 350/340 (A) | 360/360 (B) | -/- | -/- | -/- | EST |
| **PVEST196** | 318 | 310/310 (A) | -/- | 330/310 (C) | 305/305 (A) | 305/305 (A) | -/- | -/- | -/- | -/- | -/- | -/- | EST |
| **PVEST197** | 106 | 273/273 (C) | 273/273 (C) | 273/273 (C) | 273/273 (C) | 273/273 (C) | -/- | -/- | 273/273 (C) | 273/273 (C) | -/- | 273/273 (C) | EST |
| **PVEST203** | 304 | 300/300 (B) | -/- | -/- | -/- | -/- | -/- | -/- | -/- | -/- | -/- | -/- | EST |
| **PVEST217** | 248 | 240/245 (A) | -/- | 235/235 (A) | 240/240 (A) | 240/245 (A) | 245/245 (A) | -/- | 245/245 (A) | 230/230 (A) | -/- | -/- | EST |
| **PVEST221** | 304 | 298/298 (A) | -/- | 220/220 (A) | 310/310 (A) | 220/220 (A) | -/- | -/- | 310/310 (B) | -/- | -/- | -/- | EST |
| **PVEST232** | 146 | 142/142 (A) | -/- | 138/138 (D) | 155/155 (A) | 138/138 (A) | -/- | 135/135 (A) | 135/135 (A) | -/- | -/- | -/- | EST |
| **PVEST233** | 155 | 158/158 (A) | -/- | 155/153 (A) | -/- | 155/155 (A) | 158/158 (A) | -/- | -/- | -/- | -/- | -/- | EST |
| **PVEST234** | 396 | 400/400 (A) | -/- | 155/154 (A) | 400/400 (A) | 360/360 (A) | 400/400 (A) | -/- | 400/400 (B) | 360/360 (A) | -/- | -/- | EST |
| **PVEST249** | 141 | 142/142 (A) | -/- | 155/155 (A) | -/- | -/- | -/- | -/- | -/- | -/- | -/- | -/- | EST |
| **PVEST251** | 146 | 150/150 (A) | -/- | 155/156 | 158/158 (A) | 150/150 (A) | -/- | -/- | -/- | 158/158 (A) | 168/168 (A) | 158/158 (A) | EST |
| **PVEST258** | 229 | 225/225 (B) | -/- | 155/157 (B) | 185/185 (B) | 225/230 (B) | -/- | -/- | -/- | -/- | -/- | -/- | EST |
| **PVEST259** | 210 | 208/208 (A) | -/- | 155/158 | 215/215 (A) | 208/208 (A) | 220/220 (A) | -/- | -/- | -/- | -/- | -/- | EST |
| **PVEST260** | 153 | 135/135 (A) | -/- | 125/135 (A) | 135/135 (A) | 135/135 (A) | 125/125 (A) | 125/125 (A) | 135/135 (A) | 125/135 (A) | 135/135 | -/- | EST |
| **PVEST271** | 165 | 170/175 (A) | -/- | 155/160 (C) | -/- | 220/220 (B) | -/- | 340/340 (B) | 340/340 (B) | -/- | -/- | -/- | EST |
| **PVEST272** | 165 | 150/150 (A) | -/- | 150/150 (A) | 140/140 (A) | 150/150 (A) | 148/148 (A) | -/- | -/- | 140/140 (A) | 140/140 | -/- | EST |
| **PVEST293** | 192 | 180/178 (A) | -/- | 155/162 (A) | 182/182 (A) | 185/185 (A) | -/- | -/- | -/- | -/- | -/- | -/- | EST |
| **PVEST304** | 235 | 240/240 (A) | -/- | 155/163 (C) | -/- | 225/225 (C) | -/- | -/- | -/- | -/- | -/- | -/- | EST |
| **PVEST320** | 332 | 400/400 (A) | -/- | 155/164 (A) | 360/360 (A) | 360/380 (A) | 380/380 (A) | 360/360 (A) | 360/360 (A) | 340/340 (A) | -/- | -/- | EST |
| **PVEST336** | 332 | 350/350 (A) | -/- | 155/165 (C) | 348/350 (A) | 340/340 (C) | 340/340 (C) | 350/350 (C) | 340/340 © | 330/330 (C) | -/- | -/- | EST |
| **PVEST359** | 146 | 150/148 (A) | -/- | 155/166 (A) | -/- | 148/145 (A) | -/- | -/- | -/- | -/- | -/- | -/- | EST |
| **PVEST368** | 250 | 218/218 (A) | -/- | 155/167 (A) | 200/200 (A) | 178/180 (A) | 218/218 (A) | 218/218 (A) | 218/218 (A) | -/- | -/- | -/- | EST |
| **X04001** | 164 | 180/180 (A) | 165/165 (A) | 155/168 (A) | 165/165 (A) | 178/178 (A) | -/- | 160/160 (A) | 162/162 (A) | -/- | -/- | -/- | EST |
| **X13329** | 139 | 148/148 (A) | -/- | 155/169 (A) | 132/132 (A) | 143/143 (A) | 130/129 (A) | 129/129 (A) | 130/130 (A) | -/- | -/- | -/- | EST |
| **X60000** | 139 | 140/145 (A) | -/- | 155/170 (A) | -/- | 143/143 (A) | -/- | -/- | -/- | -/- | -/- | -/- | EST |

**^1^** A: strong band pattern/specific; B: weak band patterns/specific; C: strong band pattern/non-specific; D: weak band patterns/non-specific.
