# Supplementary figures and images for: The characterization of a new set of EST-derived simple sequence repeat (SSR) markers as a resource for the genetic analysis of Phaseolus vulgaris
Source: BMC Genet. 2011 May 9;12:41. doi: 10.1186/1471-2156-12-41 (PMC3102039; doi:10.1186/1471-2156-12-41)

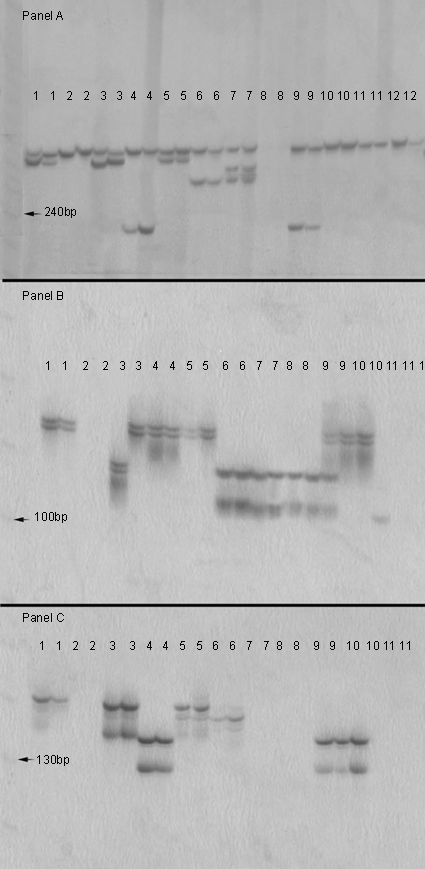

Supplement: Additional File 3 — Figure S1. Transferability of SSRs across species of the Leguminosae family. The electrophoretic pattern at BM98 (Panel A), PVEST260 (Panel B) and PVEST272 (Panel C) revealed on a polyacrylamide gels across species of the Leguminosae family: 1- Phaseolus vulgaris; 2 - Medicago sativa; 3- P. lunatus; 4- P. coccineus; 5- P. acutifolius; 6- Vigna mungo; 7- V. angularis; 8- V. unguiculata; 9- Glicine max; 10- Arachis hypogaea; 11- Dipteryx alata. [file 1471-2156-12-41-S3.PNG]

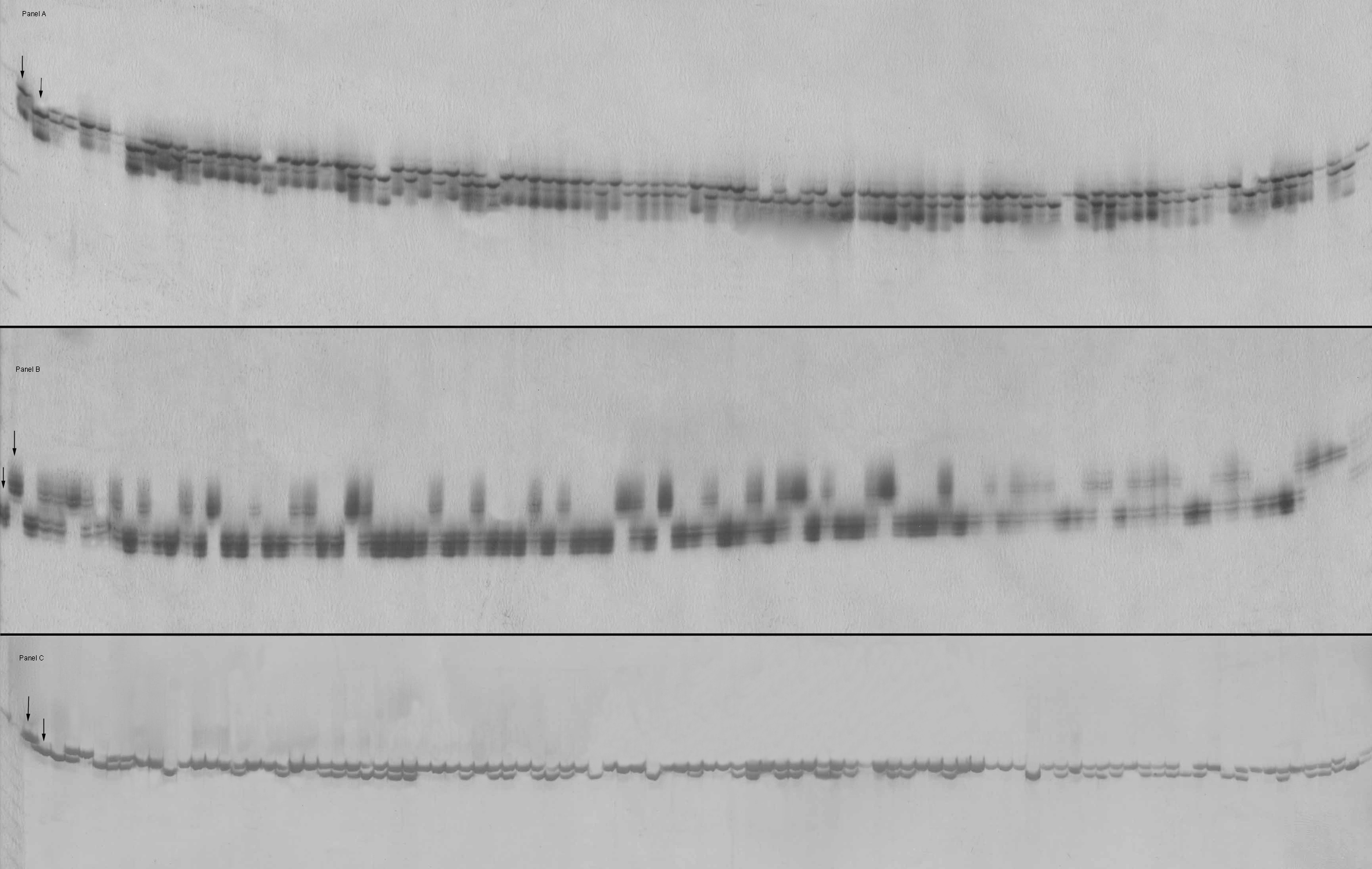

Supplement: Additional File 4 — Figure S2. Segregation of informative SSR loci in Phaseolus vulgaris. Polyacrylamide gel resolution and detection by silver staining of SSR PVEST272 (Panel A), PVEST279 (Panel B) and PVEST336 (Panel C) in BAT93 × Jalo EEP558 population. Lane 1 is a 100 bp ladder size standard with the sizes of some fragments indicated in base pairs; lanes 2 and 3 are the two parents, indicated by the arrows, followed by the segregant population. [file 1471-2156-12-41-S4.PNG]
